# Computed tomography radiomics models of tumor differentiation in canine small intestinal tumors

**DOI:** 10.3389/fvets.2024.1450304

**Published:** 2024-09-23

**Authors:** Jeongyun Jeong, Hyunji Choi, Minjoo Kim, Sung-Soo Kim, Jinhyong Goh, Jeongyeon Hwang, Jaehwan Kim, Hwan-Ho Cho, Kidong Eom

**Affiliations:** ^1^Department of Veterinary Medical Imaging, College of Veterinary Medicine, Konkuk University, Seoul, Republic of Korea; ^2^Shine Animal Medical Center, Seoul, Republic of Korea; ^3^VIP Animal Medical Center, Seoul, Republic of Korea; ^4^Daegu Animal Medical Center, Daegu, Republic of Korea; ^5^Busan Jeil Animal Medical Center, Busan, Republic of Korea; ^6^Helix Animal Medical Center, Seoul, Republic of Korea; ^7^Department of Electronics Engineering, Incheon National University, Incheon, Republic of Korea

**Keywords:** clinical radiomics models, multinomial logistic regression, random forest, support vector machine models, canine, adenocarcinoma, lymphoma, spindle cell sarcoma

## Abstract

Radiomics models have been widely exploited in oncology for the investigation of tumor classification, as well as for predicting tumor response to treatment and genomic sequence; however, their performance in veterinary gastrointestinal tumors remains unexplored. Here, we sought to investigate and compare the performance of radiomics models in various settings for differentiating among canine small intestinal adenocarcinoma, lymphoma, and spindle cell sarcoma. Forty-two small intestinal tumors were contoured using four different segmentation methods: pre- or post-contrast, each with or without the inclusion of intraluminal gas. The mesenteric lymph nodes of pre- and post-contrast images were also contoured. The bin settings included bin count and bin width of 16, 32, 64, 128, and 256. Multinomial logistic regression, random forest, and support vector machine models were used to construct radiomics models. Using features from both primary tumors and lymph nodes showed significantly better performance than modeling using only the radiomics features of primary tumors, which indicated that the inclusion of mesenteric lymph nodes aids model performance. The support vector machine model exhibited significantly superior performance compared with the multinomial logistic regression and random forest models. Combining radiologic findings with radiomics features improved performance compared to using only radiomics features, highlighting the importance of radiologic findings in model building. A support vector machine model consisting of radiologic findings, primary tumors, and lymph node radiomics features with bin count 16 in post-contrast images with the exclusion of intraluminal gas showed the best performance among the various models tested. In conclusion, this study suggests that mesenteric lymph node segmentation and radiological findings should be integrated to build a potent radiomics model capable of differentiating among small intestinal tumors.

## 1 Introduction

The full workflow of radiomics involves various steps, including clinical data collection, image acquisition and preprocessing, image segmentation or target region delineation, feature extraction and selection, and model building (1). All these steps can significantly affect the results of the radiomics analysis, meticulous studies of each step are performed. For instance, acquiring an adequate number of image samples is important because numerous radiomic features are analyzed (1, 2). Various imaging parameters and protocols among institutions have been found to increase feature instability. Image acquisition factors, including tube current and slice thickness, have been thoroughly studied in human radiomics (3–5). Image preprocessing includes registration, filtering, and intensity normalization (6); image registration aligns multimodal images; filtering reduces noise; and normalization stabilizes feature extraction (7–9). Image segmentation can be performed manually, semi-automatically, or automatically, and comparisons among these methods have been performed (10). Manual segmentation utilizes expert knowledge—yet, inter- and intra-observer variability does exist. By contrast, semi-automatic segmentation allows the user to modify algorithm-created segmented regions, whereas automatic segmentation uses computer-based segmentation (11, 12).

Most radiomics feature-extraction studies involve the generation and assessment of histograms, textures, and morphological features (13, 14). Histogram features represent Hounsfield unit (HU) intensity, and texture features quantify tumor heterogeneity by typically including gray-level co-occurrence and gray-level size zone matrices (15). Morphological features show tumor shapes in two- and three-dimensional images. The effects of the feature extraction method, including bin sizes and numbers, have also been vigorously studied (16). Recently, artificial-intelligence convolutional neural networks were adopted to increase harmonization, feature stability, and reliability (17). Feature selection deploys filter, wrapper, embedded, and dimension-reduction methods (18) to select crucial features from among the numerous features calculated. Least absolute shrinkage and selection operator (LASSO), as well as ridge regression, are embedded methods commonly used in radiomics (19). Model building in clinical radiomics settings can aid diagnosis, provide prognosis, and predict responses (20). Common models used are support vector machine, random forest, linear discriminant analysis, and multinomial logistic regression (21–24).

The role of radiomics models in human oncology has been widely investigated. Studies have not only investigated tumor classification and genomic sequence prediction but also focused on predicting tumor response after treatment (20, 25–30). Researches have demonstrated the role of radiomics models in predicting the response to bevacizumab in brain necrosis following radiotherapy (31), as well as their promising potential for predicting lung tumor shrinkage during chemoradiation (30, 32), for the diagnosis and prediction of nasopharyngeal carcinoma (20), and their diagnostic significance for various tumors.

Conversely, in veterinary radiomics, few studies have investigated the role of radiomics models in canine malignancies. A study by Banzato et al. utilized linear discriminant analysis and identified the most discriminant factor to predict the grade of canine meningioma (33). Another study used multiple quadratic discriminant models to differentiate between benign and malignant liver tumors (34). Linear discriminant analysis has also been performed to differentiate between benign and malignant splenic tumors (35).

Few studies have investigated the role of radiomics models of gastrointestinal tract tumors in humans, with a main focus on gastrointestinal stromal cell tumors, a subtype of spindle cell sarcoma (36–40). However, no studies have been conducted on radiomics models of veterinary gastrointestinal tumors. Thus, the objective of this study was to investigate various models and their performance in differentiating between small intestinal adenocarcinoma, lymphoma, and spindle cell sarcoma.

## 2 Materials and methods

### 2.1 Ethics statements

This study was a retrospective investigation, and ethical review and approval were not required. It was approved by each participating institution.

### 2.2 Data collection

This multi-institutional study included cases from six animal medical centers: Konkuk Veterinary Teaching Hospital, VIP Animal Medical Center, Shine Animal Medical Center, Helix Animal Medical Center, Daegu Animal Medical Center, and Busan Jeil Animal Medical Center. Dogs diagnosed with small intestinal tumors were investigated. Examinations conducted between 2012 and 2022 were reviewed. The data from multiple institutions were compiled and evaluated by a single veterinarian (JJ). The inclusion criteria were: (1) histopathologic diagnosis of intestinal adenocarcinoma, lymphoma, spindle cell sarcoma, or cytologic diagnosis of small intestinal lymphoma and (2) computed tomography (CT) examinations of intestinal tumors. The exclusion criteria were as follows: (1) absence of a prominent small intestinal mass on CT examination and (2) ileocecocolic junction mass, the exact origin of which could not be determined on CT examination (ileum, cecum, and colon).

### 2.3 Computed tomographic examinations

CT examinations were performed using LightSpeed (General Electric Medical System, Chicago, IL, United States), Revolution ACT (General Electric Medical System), Brivo CT 385 (General Electric Medical System), Aquilion (Canon Medical Corporation, Tochigi, Japan), Aquilion Lightning (Canon Medical Corporation), BrightSpeed (General Electric Medical System), and Somatom Scope (Siemens Healthcare, Erlangen, Germany). The number of CT slices ranged from 4 to 64. CT acquisition settings included a slice thickness of 0.8–2.5 mm, helical pitch of 0.70–1.75 mm, and matrix dimension of 512 × 512 with variable fields of view. The detailed acquisition settings for the scanners are listed in Supplementary Table S1. The patients were placed in either the supine or prone position under general anesthesia. Owing to the retrospective nature of the data, detailed contrast medium dosages, injection speeds, or anesthesia protocols were not included in all examinations. For cases with the aforementioned records, nonionic contrast medium (iohexhol 350 mg/mL; Onimpaque, GE Healthcare, Princeton, NJ, United States) was administered via a power injector at a rate of 2.0–2.5 mL/s or manually. Post-contrast CT images were obtained during the portal phase, approximately between 60 and 90 s after contrast medium injection. All the CT images were saved as Digital Imaging and Communication in Medicine.

### 2.4 Image segmentation

Small intestinal tumor segmentation was performed using the commercially available software 3D Slicer.^1^ Manual segmentation of the intestinal tumors in both pre- and post-contrast CT images was performed. Where the intestinal tumor margin was unclear on pre-contrast CT images, post-contrast CT images and their segmented areas were used as references. The segmented tumor region of interest was drawn by one radiologist with 4 years of experience (JJ) and confirmed by two senior radiologists (JK and KE). When there were different opinions in terms of tumor segmentation, final segmentation was made based on consensus.

**Figure 1:**
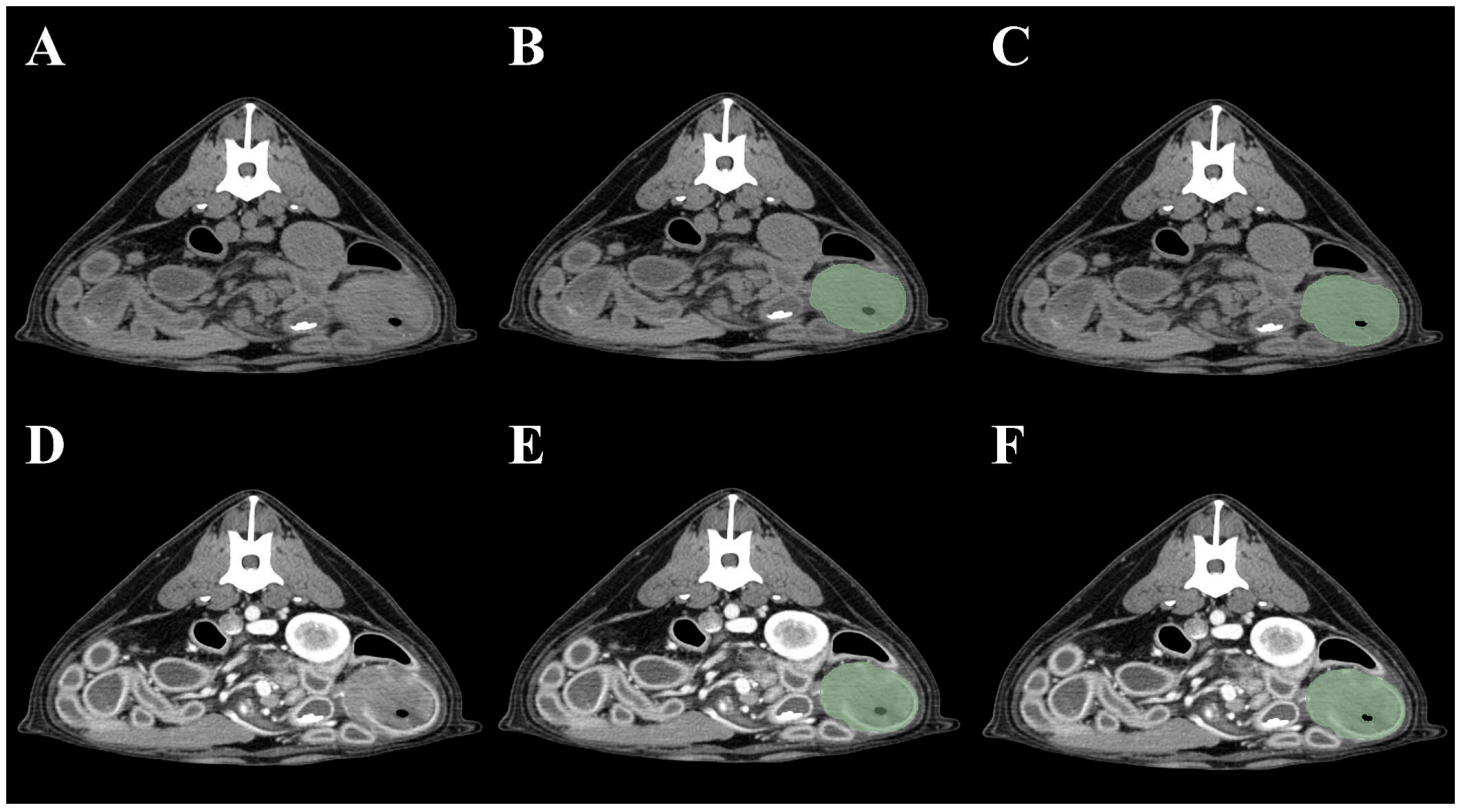
Four methods of intestinal segmentations in pre-contrast **(A)** and post-contrast images **(D)**. Method 1 **(B)**: pre-contrast, gas-included segmentation; Method 2 **(E)**: post-contrast, gas-included segmentation; Method 3 **(C)**: pre-contrast, gas-excluded segmentation; Method 4 **(F)**: post-contrast, gas excluded-segmentation.

As the small intestine is a hollow organ containing intraluminal gas, two contouring methods were devised. The first included the intraluminal gas, whereas the second did not. To exclude gas in the second method, a threshold cutoff of −150 HU was set so that pixels <−150 HU would not be selected. Thus, four segmentation methods were created for each intestinal tumor: (1) pre-contrast, gas included; (2) post-contrast, gas included; (3) pre-contrast, gas excluded; and (4) post-contrast, gas excluded. Method 1 was defined as pre-contrast, gas-included segmentation; Method 2 as post-contrast, gas-included segmentation; Method 3 as pre-contrast, gas-excluded segmentation; and Method 4 as post-contrast, gas-excluded segmentation (Figure 1). Mesenteric lymph nodes were also segmented to investigate whether the presence of mesenteric lymph node radiomics feature data contributed to the radiomics model performance. When multiple mesenteric lymph nodes were identified, the lymph node that best represented the overall status of the mesenteric lymph nodes was selected by a radiologist (JJ). Both pre- and post-contrast lymph node images were segmented.

### 2.5 Qualitative computed tomographic evaluation

All CT images were reviewed using commercially available software (Radiant, Medixant, Poznan, Poland) by a radiologist (JJ) under the supervision of two senior radiologists (JK and KE). CT images were displayed in soft-tissue and lung-window settings [soft-tissue window (window level, 60; window width, 400) and lung window (window level, −400; window width, 1,500)]. The evaluation criteria for intestinal tumors were established similarly to previous studies (41, 42) and included: location (duodenum, jejunum, ileum), tumor growth pattern (concentric, eccentric, mixed), margin (well-defined or poorly defined), obstruction (present or absent), mineralization (present or absent), mesentery fat stranding (present or absent), peritoneal effusion (present of absent), mesenteric lymphadenopathy (present or absent), metastases in thoracic or abdominal organs (present or absent), enhancement pattern (homogenous or heterogenous). Obstruction was considered present when the maximum diameter of the tumor was greater than the height of the L5 vertebral body (43). Fat stranding was defined as a misty appearance or increased attenuation of the mesenteric fat (44), and lymphadenopathy was considered present if the size was >5 mm in any dimension (45).

### 2.6 Radiomics feature extraction and model building

Tumor and lymph node radiomics features were computed through various bin settings using Pyradiomics (Python-based) (13). The bin settings included bin counts (BC) and widths (BW) of 16, 32, 64, 128, and 256. Seventy-two radiomics features (14 shape features, 18 histogram features, 24 gray level co-occurrence matrix, and 16 gray level size zone matrix) were computed. Detailed information on these features is provided in Supplementary Table S2.

Radiomics models were constructed using the Statistics and Machine Learning Toolbox in MATLAB (MathWorks, Natick, MA, United States). Feature selection and training/test set formation for model building were chosen by multinomial LASSO with 0.3 holdout ratio and 100 repetitions. Multinomial logistic, random forest, and support vector machine (SVM) models were developed. All models were built for segmentation of tumors only or tumors and mesenteric lymph nodes. Models built using tumor segmentation only were termed “Primary Tumor (PT) models,” whereas those built using tumor and lymph node segmentations were termed “Primary Tumor and Lymph Node (PTLN) models.” Tumors in pre- and post-contrast images were matched with lymph nodes in pre- and post-contrast images, respectively. Models were built for all bin settings and segmentation methods.

In addition to the radiomics models, qualitative CT findings were added as features to build clinical-radiomics models. Clinical-radiomics models were constructed using the Statistics and Machine Learning Toolbox in MATLAB. Multinomial LASSO with 0.3 holdout ratio of 100 times repetition was used to select features and divide training/test set. Random forest and SVM models were created. Similar to the radiomics models, the PT and PTNL clinical-radiomics models were built to combine radiological findings with radiomics features for all bin settings and segmentation methods.

### 2.7 Statistical analyses

All statistical analyses were performed using the Statistics and Machine Learning Toolbox in MATLAB. For all the models, the accuracy and area under the curve (AUC) of the training and test sets were calculated. The AUCs for the training and test sets were calculated as the mean of the model prediction performance for the individual tumor subtypes (adenocarcinoma, lymphoma, and spindle cell sarcoma). The DeLong test was used to compare the performance of the models.

For PTLN models, a method with the highest performance was analyzed for commonly selected features, whereas for the PTLN clinical-radiomics models, a method with the highest performance was analyzed for both commonly selected features and radiological findings. Among the 100 bootstrapping repetitions, radiomics features and radiologic findings selected >95 times were regarded as commonly selected. *T*-tests were used to compare commonly selected radiomic features of small intestinal adenocarcinoma, lymphoma, and spindle cell sarcoma. Fisher’s exact tests were used to compare commonly selected radiological findings among the tumors. A *p*-value of <0.05 denoted statistical significance.

## 3 Results

### 3.1 Patient population

In total, 41 dogs met the inclusion criteria. The mean age was 11.3 years (range, 5–18 years) and the mean body weight was 7.5 kg (range, 1.9–37.0 kg). Sixteen castrated males, 19 spayed females, one intact male, and five intact females were included. The following breeds were included: Shiba Inu (1), Dachshund (2), Pomeranian (2), Poodle (5), Cane Corso (1), Miniature Pinscher (2), Maltese (12), Schnauzers (2), Pug (1), Yorkshire Terrier (3), Beagle (2), Rottweiler (1), Spitz (1), Shih Tzu (1), Bichon Frise (1), Cocker Spaniel (1), Shetland Sheepdog (1), Pungsan (1), and Mixed (1). Forty-two intestinal tumors were segmented, including nine adenocarcinomas, 14 lymphomas, and 19 spindle cell sarcomas. All adenocarcinomas and spindle-cell sarcomas were diagnosed via histopathological examination. Four lymphoma cases were diagnosed by cytological examination while 10 cases were diagnosed by histopathological examination. One dog with lymphoma had two multifocal intestinal masses that were both diagnosed as lymphomas. Each intestinal mass was considered an individual intestinal tumor case. Different mesenteric lymph nodes were segmented for each tumor. The patient demographics are presented in Supplementary Table S3.

### 3.2 Computed tomography features

Adenocarcinomas included one tumor in the duodenum, five tumors in the jejunum, and three tumors in the ileum. Lymphomas included two tumors in the duodenum, 10 tumors in the jejunum and two tumors in the ileum. Spindle cell sarcomas consisted of three tumors in the duodenum, 15 tumors in the jejunum, and one tumor in the ileum. In terms of growth patterns, adenocarcinomas showed two concentric, one eccentric, and six mixed patterns. Lymphomas exhibited 10 concentric and four eccentric patterns. Spindle cell sarcomas demonstrated 14 eccentric and five mixed patterns. The adenocarcinoma margins were well-defined in seven cases and poorly defined in two cases. Lymphomas included 10 well-defined margins and four poorly defined margins. Spindle cell sarcomas showed well-defined margins in 18 cases and poorly defined margins in one case. Obstruction was noted in four cases of adenocarcinoma, one case of lymphoma, and two cases of spindle cell sarcoma. Mineralization was present in one case of adenocarcinoma, two cases of lymphoma, and three cases of spindle cell sarcoma. Fat stranding was present in six cases of adenocarcinomas, six cases of lymphomas and four cases of spindle cell sarcoma. Peritoneal effusion was noted in one case of adenocarcinoma, two cases of lymphomas and three cases of spindle cell sarcoma. Lymphadenopathy was noted in four cases of adenocarcinoma, 12 cases of lymphomas and two cases of spindle cell sarcoma. Metastases were observed in one case of adenocarcinoma and two cases of spindle cell sarcoma. A heterogeneous enhancement pattern was noted in eight adenocarcinomas and 18 spindle-cell sarcomas. Homogenous enhancement was observed in all lymphomas. The results are summarized in Table 1.

**Table 1:** Summary of qualitative CT analysis of intestinal tumors.

		Adenocarcinoma (*n* = 9)	Lymphoma (*n* = 14)	Spindle cell sarcoma (*n* = 19)
Location	Duodenum	1/9	2/14	3/19
	Jejunum	5/9	10/14	15/19
	Ileum	3/9	2/14	1/19
Growth pattern	Concentric	2/9	10/14	0/19
	Eccentric	1/9	4/14	14/19
	Mixed	6/9	0/14	5/19
Margin	Well-defined	7/9	10/14	18/19
	Ill-defined	2/9	4/14	1/19
Obstruction	Present	4/9	1/14	2/19
	Absent	5/9	13/14	17/19
Mineralization	Present	1/9	2/14	3/19
	Absent	8/9	12/14	16/19
Fat stranding	Present	6/9	6/14	4/19
	Absent	3/9	8/14	15/19
Peritoneal effusion	Present	1/9	2/14	3/19
	Absent	8/9	12/14	16/19
Lymphadenopathy	Present	4/9	12/14	2/19
	Absent	5/9	2/14	17/19
Metastases	Present	1/9	0/14	2/19
	Absent	8/9	14/14	17/19
Enhancement pattern	Homogenous	1/9	14/14	1/19
	Heterogenous	8/9	0/14	18/19

### 3.3 Model performance of multinominal logistic, random forest, and SVM radiomics models

The accuracy and AUC for the training and test sets of the PT multinomial logistic models are summarized in Supplementary Tables S4, S5. Among the BC settings, Method 4 with BC32 exhibited the highest test accuracy (0.6583) and AUC (0.8224). Method 2 with BC128 showed the lowest test accuracy (0.5092) and AUC (0.6973). Among the BW settings, Method 2 with BW16 exhibited the highest test accuracy (0.6342), and Method 4 with BW16 exhibited the highest test AUC (0.8082). Method 3 with BW16 and BW32 had the lowest test accuracy (0.4992), whereas Method 1 with BW16 demonstrated the lowest AUC (0.6763).

For the PTLN multinomial logistic models, the accuracies and AUC for the training and test sets are presented in Supplementary Tables S6, S7. Among the BC settings, Method 4 with BC16 exhibited the highest test accuracy (0.7592), and Method 4 with BC32 had the highest test AUC (0.8881). Method 3 with BC16 exhibited the lowest test accuracy (0.6650) and AUC (0.8294). Among the BW settings, Method 4 with BW16 had the highest test accuracy (0.7758) and AUC (0.9034). Method 4 with BW256 exhibited the lowest test accuracy (0.6775), and Method 3 with BW1, exhibited the lowest test AUC (0.8360).

For the PT random forest models, the accuracy and AUC for the training and test sets are summarized in Supplementary Tables S8, S9. Among the BC settings, Method 1 with BC16 showed the highest test accuracy (0.6052) and AUC (0.7728). Method 3 with BC16 exhibited the lowest test accuracy (0.4867), and Method 2 with BC64 exhibited the lowest test AUC (0.6853). Among the BW settings, Method 1 with BW16 had the highest test accuracy (0.5925), and Method 4 with BW16 had the highest test AUC (0.7797). Method 2 with BW32 exhibited the lowest test accuracy (0.4892), and Method 2 with BW256 had the lowest test AUC (0.6631).

The accuracies and AUC for the training and test sets of the PTLN random forest models are presented in Supplementary Tables S10, S11. Among the BC settings, Method 1 with BC256 had the highest test accuracy (0.7858), and Method 4 with BC32 had the highest test AUC (0.9231). Method 2 with BC256 exhibited the lowest test accuracy (0.7267), whereas Method 3 with BC16 had the lowest test AUC (0.8767). Among the BW settings, Method 1 with BW64 demonstrated the highest test accuracy (0.7833), and Method 1 with BW32 showed the highest test AUC (0.8927). Method 2 with BW128 exhibited the lowest test accuracy (0.6842), and Method 3 with BW256 exhibited the lowest test AUC (0.8495).

The accuracy and AUC for the training and test sets of the PT SVM models are summarized in Supplementary Tables S12, S13. Among the BC settings, Method 4 with BC16 showed the highest test accuracy (0.7600), and Method 4 with BC32 exhibited the highest test AUC (0.8875). Method 2 with BC128 had the lowest test accuracy (0.6067), and Method 3 with BC128 had the lowest test AUC (0.7685). Among the BW settings, Method 2 with BW16 showed the highest test accuracy (0.6967) and AUC (0.8625). Method 2 with BW128 had the lowest test accuracy (0.5733), and Method 2 with BW256 exhibited the lowest test AUC (0.7490).

The accuracies and AUC for the training and test sets of the PTLN SVM models are presented in Supplementary Tables S14, S15. Among the BC settings, Method 3 with BC32 demonstrated the highest test accuracy (0.8067), and Method 4 with BC32 had the highest test AUC (0.9360). Method 2 which used BC128 exhibited the lowest test accuracy (0.7100) and AUC (0.8715). Among the BW settings, Method 3 with BW128 had the highest test accuracy (0.8225) and AUC (0.9483). Method 3 with BW16 exhibited the lowest test accuracy (0.7167) and AUC (0.8760).

### 3.4 Model performance of random forest, SVM clinical-radiomics models

For the PT random forest clinical-radiomics models, the accuracy and AUC for the training and test sets are summarized in Supplementary Tables S16, S17. Among the BC settings, Method 4 with BC32 showed the highest test accuracy (0.7633) and AUC (0.9200). Method 3 with BC256 had the lowest test accuracy (0.6675), and Method 2 with BC32 had the lowest test AUC (0.8352). Among the BW settings, Method 1 with BW256 exhibited the highest test accuracy (0.7592), and Method 4 with BW16 had the highest test AUC (0.9033). Method 4 with BW128 exhibited the lowest test accuracy (0.6733), and Method 2 with BW32 exhibited the lowest test AUC (0.8523).

For the PTLN random forest clinical-radiomics models, the accuracy and AUC for the training and test sets are presented in Supplementary Tables S18, S19. Among the BC settings, Method 1 with BC16 demonstrated the highest test accuracy (0.7925), and Method 4 with BC32 showed the highest test AUC (0.9419). Method 2 with BC32 exhibited the lowest test accuracy (0.7333), and Method 3 with BC256 exhibited the lowest test AUC (0.8965). Among the BW settings, Method 1 with BW64 had the highest test accuracy (0.8200) and AUC (0.9207). Method 4 with BW128 exhibited the lowest test accuracy (0.7150), and Method 3 with BW16 exhibited the lowest test AUC (0.8983).

For the PT SVM clinical-radiomics models, the accuracy and AUC for the training and test sets are summarized in Supplementary Tables S20, S21. Among the BC settings, Method 4 with BC16 had the highest test accuracy (0.9058) and AUC (0.9770). Method 2 with BC16 exhibited the lowest test accuracy (0.6983) and AUC (0.8574). Among the BW settings, Method 1 with BW256 showed the highest test accuracy (0.7750), and Method 4 with BW16 exhibited the highest test AUC (0.8905). Method 2 with BW32 and BW256 exhibited the lowest test accuracy (0.6892), and Method 1 with BW32 exhibited the lowest test AUC (0.8429).

For the PTLN SVM clinical-radiomics models, the accuracy and AUC for the training and test sets are presented in Supplementary Tables S22, S23. Among the BC settings, Method 4 with BC32 showed the highest test accuracy (0.9050), and Method 4 with BC16 showed the highest test AUC (0.9775). Method 2 with BC256 had the lowest test accuracy (0.7975) and AUC (0.9311). Among the BW settings, Method 4 with BW16 demonstrated the highest test accuracy (0.8567), and Method 1 with BW64 showed the highest test AUC (0.9565). Method 4 with BW256 exhibited the lowest test accuracy (0.7792), and Method 4 with BW128 exhibited the lowest test AUC (0.9134).

### 3.5 Performance comparison of PT and PTLN models

**Figure 2:**
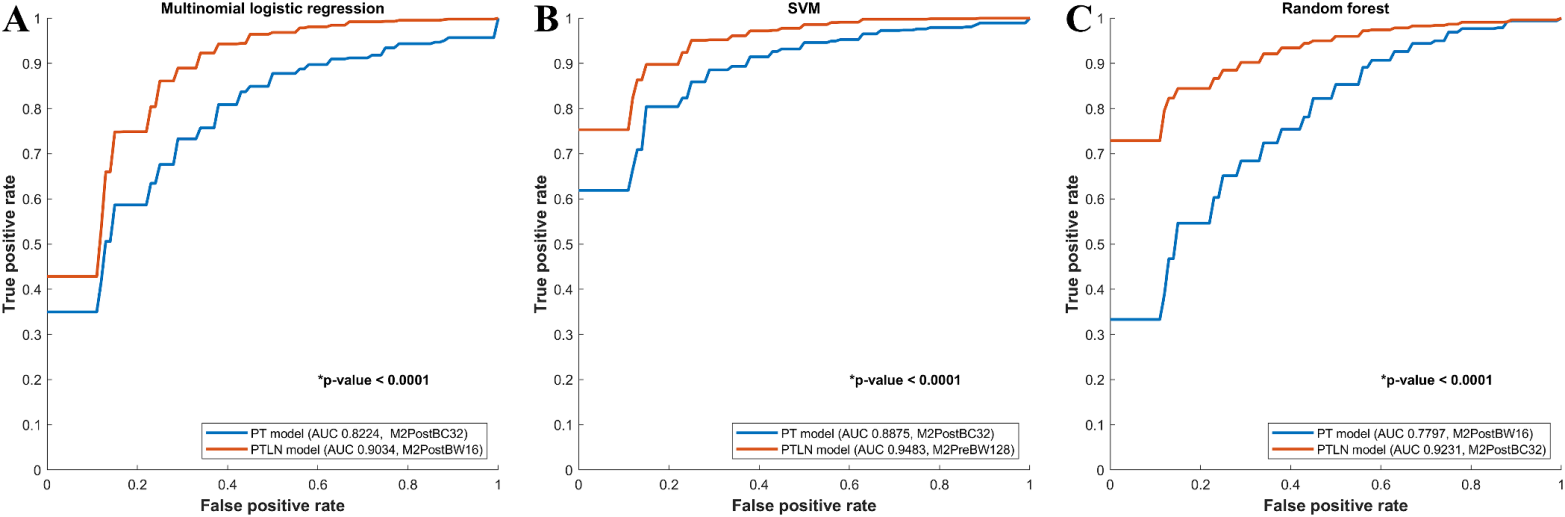
Comparison between receiver-operating characteristics curves of the PT and PTLN in multinomial logistic regression, random forest, and SVM models. **(A)** The PTLN model using Method 4 and BW16 showed a significantly higher AUC than the PT model using Method 4 and BC32 (AUC 0.9034 vs. 0.8224). **(B)** The PTLN model using Method 4 and BC32 demonstrated a significantly higher AUC than the PT model using Method 4 and BW16 (AUC 0.9231 vs. 0.7797). **(C)** The TLN model using Method 3 and BW128 exhibited a significantly higher AUC than the PT model with method 4 and BC32 (AUC 0.9483 vs. 0.8875).

In the multinomial logistic regression models, the PT and PTLN models with the best AUC performance were compared. The PTLN model using Method 4 and BC32 showed a significantly higher AUC than the PT model using Method 4 and BW16 (AUC 0.9034 vs. 0.8224) (Figure 2A).

Among the random forest models, the PT and PTLN models with the best AUC performance were compared. The PTLN model using Method 4 and BC32 demonstrated a significantly higher AUC than the PT model using Method 4 and BW16 (AUC 0.9231 vs. 0.7797) (Figure 2B).

In the SVM models, the PT and PTLN models with the best AUC performance were compared. The PTLN model using Method 3 and BW128 exhibited a significantly higher AUC than the PT model using Method 4 and BC32 (AUC 0.9483 vs. 0.8875) (Figure 2C).

**Figure 3:**
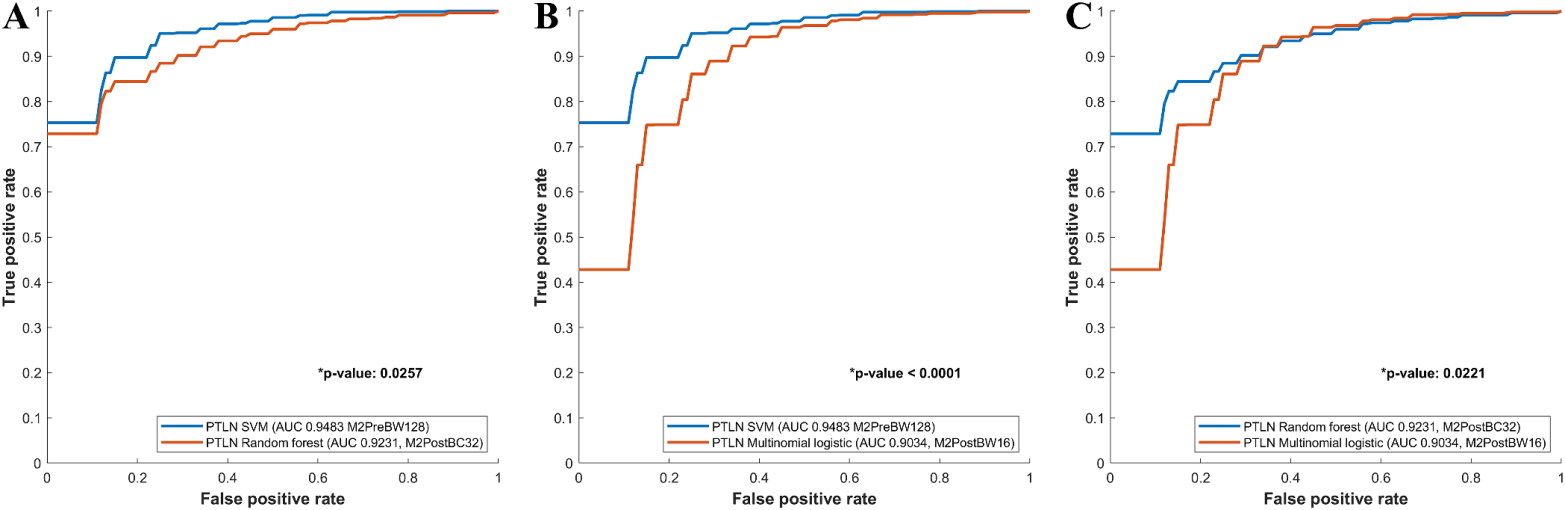
Comparison between receiver-operating characteristics curves of the PTLN multinomial logistic regression, random forest, SVM models. **(A)** The SVM model using Method 3 and BW128 exhibited a significantly higher AUC than the random forest model using Method 4 and BC32 (AUC 0.9483 vs. 0.9231). **(B)** The SVM model using Method 3 and BW128 showed a significantly higher AUC than the multinomial logistic regression model using Method 4 and BW16 (AUC 0.9483 vs. 0.9034). **(C)** The random forest model with Method 4 and BC32 exhibited a significantly higher AUC than the multinomial logistic regression model using Method 4 and BW16 (AUC 0.9231 vs. 0.9034).

The PTLN models with the best AUC performance in the multinomial logistic regression, random forest, and SVM models were compared. The SVM model using Method 3 and BW128 exhibited a significantly higher AUC than the random forest model using Method 4 and BC32 (AUC 0.9483 vs. 0.9231) (Figure 3A). The SVM model using Method 3 and BW128 showed a significantly higher AUC than the multinomial logistic regression model using Method 4 and BW16 (AUC 0.9483 vs. 0.9034) (Figure 3B). The random forest model using Method 4 and BC32 exhibited a significantly higher AUC than the multinomial logistic regression model using Method 4 and BW16 (AUC 0.9231 vs. 0.9034) (Figure 3C).

### 3.6 Performance comparison of PTLN clinical-radiomics models

PTLN clinical-radiomics models, which include radiological findings, were compared with PTLN models to investigate the effect of incorporating radiological findings.

**Figure 4:**
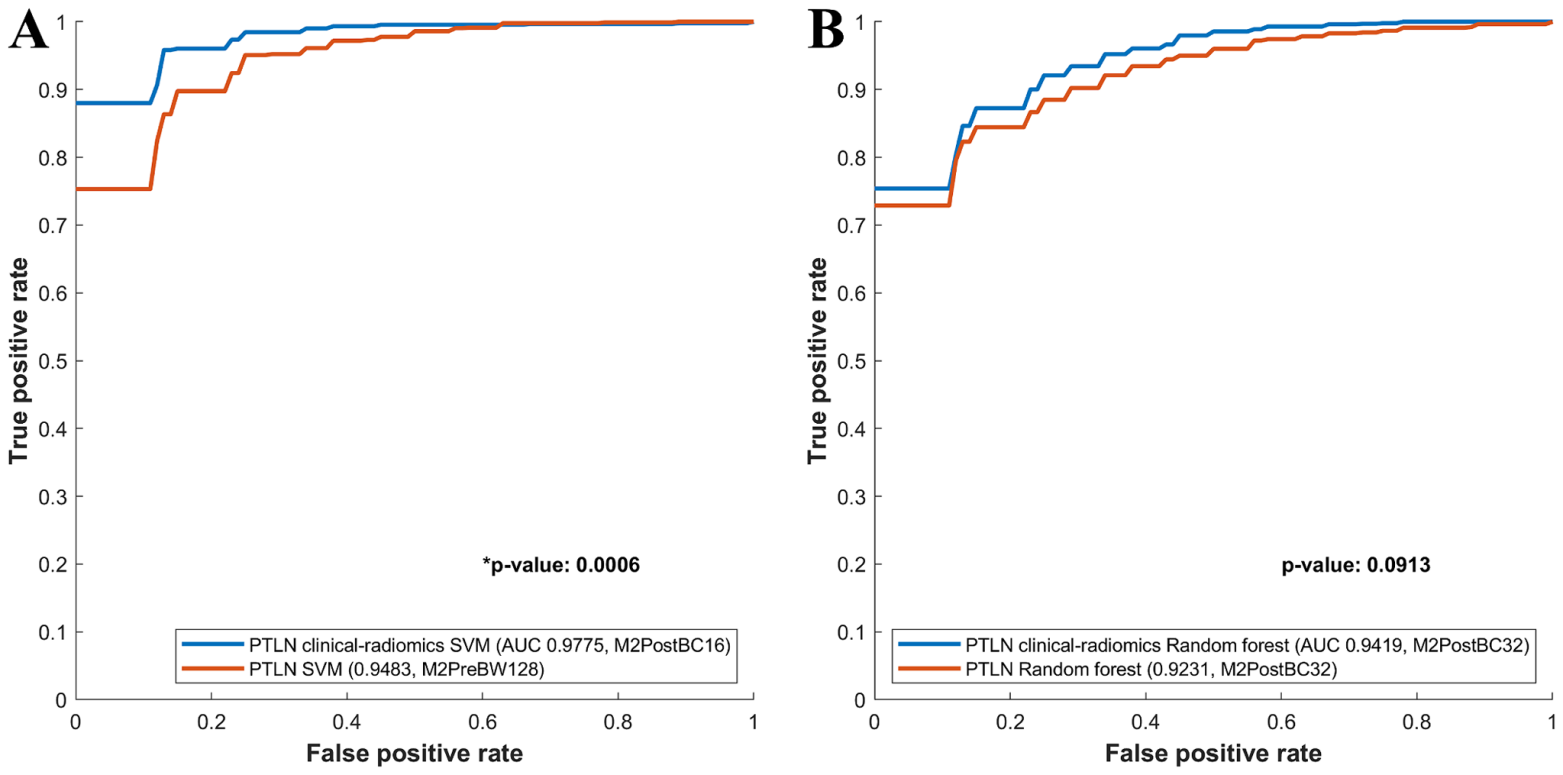
Comparison between receiver-operating characteristics curves of the PTLN clinical-radiomics SVM, random forest and PTLN SVM, random forest. **(A)** The clinical-radiomics SVM model with Method 4 and BC16 showed a significantly higher AUC than the radiomics SVM model with Method 3 and BW128 (AUC 0.9775 vs. 0.9483). **(B)** The clinical-radiomics random forest model with Method 4 and BC32 exhibited a higher AUC than the radiomics random forest model with Method 4 and BC32 (AUC 0.9419 vs. 0.9231), although the difference was not significant.

Among the PTLN SVM models, those with the best AUC performance in the clinical-radiomics and radiomics models were compared. The clinical-radiomics SVM model with Method 4 and BC16 showed a significantly higher AUC than the radiomics SVM model with Method 3 and BW128 (AUC 0.9775 vs. 0.9483) (Figure 4A).

Among the PTLN random forest models, the models with the best AUC performance in the clinical-radiomics and radiomics models were also compared. The clinical-radiomics random forest model using Method 4 and BC32 exhibited a higher AUC than the radiomics random forest model using Methods 4 and BC32 (AUC 0.9419 vs. 0.9231), although the difference was not significant (Figure 4B).

**Figure 5:**
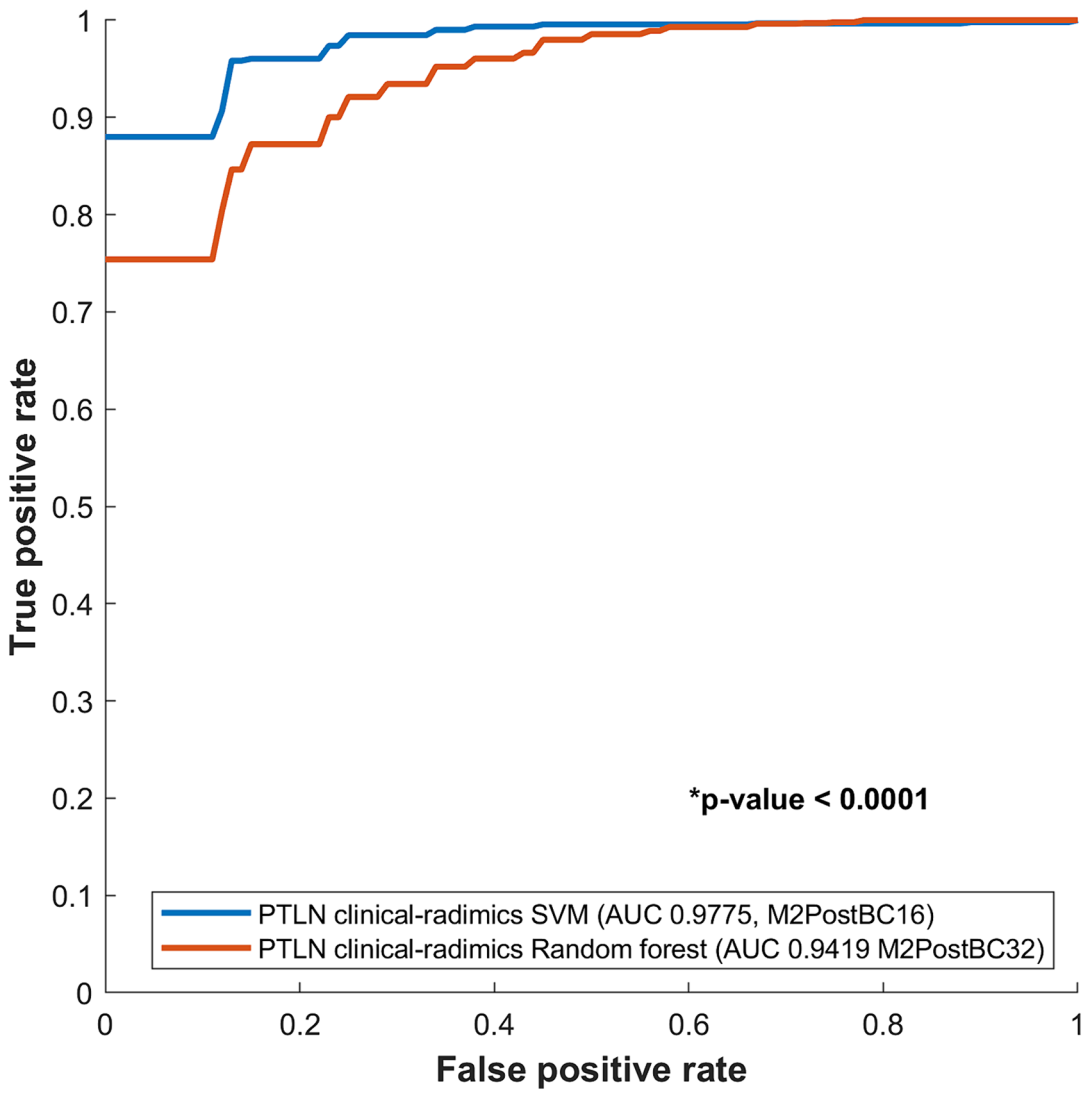
Comparison between receiver-operating characteristics curves of the PTLN clinical-radiomics SVM and random forest models. The SVM model with Method 4 and BC16 showed a significantly higher AUC than the random forest model with Method 4 and BC32 (AUC 0.9775 vs. 0.9412).

The PTLN clinical-radiomics models with the best AUC performance in random forest and SVM were compared. The SVM model with Method 4 and BC16 showed a significantly higher AUC than random forest model with Method 4 and BC32 (AUC 0.9775 vs. 0.9419) (Figure 5).

### 3.7 Commonly selected radiomics features

The PTLN and PTLN clinical-radiomics models with the best performance were analyzed for commonly selected features and radiological findings. For the PTLN models, SVM Method 3 with BW128, and for the PTLN clinical-radiomics models, SVM Method 4 with BC16, showed the best performance.

For PTLN models, commonly selected features among 100 times of bootstrapping in Method 3 with BW128 included elongation, LeastAxis Length, SurfaceVolumeRatio, LowGrayLevelZoneEmphasis for tumor segmentation and Maximum2DDiameterSlice, Kurtosis, and Minimum for lymph node segmentation.

For PTLN clinical-radiomics, commonly selected features among 100 times of bootstrapping in Method 4 with BC16 included LeastAxisLength, SurfaceVolumeRatio, Kurtosis for tumor segmentation and Maximum2DDiameterSlice, and InterquartileRange for lymph node segmentation. Radiological findings commonly included growth patterns, obstruction, and fat stranding.

### 3.8 Comparison among adenocarcinoma, lymphoma, and spindle cell sarcoma in commonly selected radiomics features

Commonly selected radiomics features were compared among the tumor subtypes and are summarized in Table 2.

**Table 2:** Comparison among commonly selected radiomics features.

		Adenocarcinoma	Lymphoma	Spindle Cell Sarcoma	*p*-value
PTLN Model: Method 3 with BW128	Elongation	0.69 [0.42, 0.96]	0.68 [0.46, 0.91]	0.80 [0.56, 0.97]^a*^	0.013^a^
	LeastAxisLength	19.31 [11.41, 27.61]^a*^	22.01 [11.52, 47.74]	38.00 [16.31, 87.35]^b*^	0.021^a^, <0.001^b^
	SurfaceVolumeRatio	0.36 [0.23, 0.57]	0.38[0.13, 0.52]^a*^	0.21 [0.06, 0.40]^b*^	0.002^a^, <0.001^b^
	LowGrayLevelZoneEmphasis	0.68 [0.33, 0.99]	0.56 [0.23, 0.99]	0.45 [0.20, 0.99]	
	Maximum2DDiameterSlice_LN	9.38 [6.53, 16.54]	26.12 [4.86, 48.31]^a*^	8.70 [3.54, 15.49]^b*^	<0.001^a^, 0.003^b^
	Kurtosis_LN	3.74 [2.76, 5.95]	5.95 [2.61, 8.88]^a*^	2.93 [1.97, 4.51]^b*^	<0.001^a^, 0.007^b^
	Minimum_LN	−62.66 [−91, −46]^a*^	−75.28 [−146, −35]	−80.36 [−106, −59]	0.014^a^
PTLN Clinical-Radiomics Model: Method 4 with BC16	LeastAxisLength	20.44 [14.79, 27.74]^a*^	21.99 [11.82, 44.84]	38.19 [17.26, 86.29]^b*^	0.029^a^, <0.001^b^
	SurfaceVolumeRatio	0.35 [0.23, 0.45]	0.38 [0.14, 0.55]^a*^	0.20 [0.06, 0.37]^b*^	<0.001^a, b^
	Kurtosis	11.77 [2.34, 49.89]	15.73 [2.80, 67.14]	20.39 [2.47, 208.06]	
	Maximum2DDiameterSlice_LN	9.38 [6.77, 16.54]	26.12 [4.92, 48.37]^a*^	8.70 [3.41, 15.25]^b*^	<0.001^a^, 0.048^b^
	InterquartileRange_LN	27.06 [24, 70.75]	19.14 [11, 54]^a*^	36.48 [35, 89]^b*^	<0.001^a, b^

All data are presented as mean and range [minimum, maximum]. *p < 0.05 is considered statistically significant.* *p < 0.05. PTLN Primary Tumor and Lymph Node, BC, Bin Count; BW, Bin Width; LN, Lymph Node.

For Method 3, with BW128, the Elongation was significantly higher in spindle cell sarcomas than in other tumors. LeastAxisLength demonstrated a significantly higher value in spindle cell sarcoma and a significantly lower value in adenocarcinoma, whereas SurfaceVolumeRatio showed significantly higher values in lymphoma and significantly lower values in spindle cell sarcoma than in other tumors. LowGrayLevelZoneEmphasis was significantly lower in spindle cell sarcoma but not among tumors. In terms of lymph nodes, the Maximum2DDiameterSlice and Kurtosis exhibited significantly higher values for lymphoma and significantly lower values for spindle cell sarcoma than for other tumors. The minimum was significantly higher in adenocarcinomas than in other tumors.

For Method 4, with BC16, the LeastAxisLength showed a significantly higher value in spindle cell sarcoma and a significantly lower value in spindle cell sarcoma compared to other tumors. The SurfaceVolumeRatio was significantly higher in lymphomas and significantly lower in spindle cell sarcomas compared to other tumors. Kurtosis was higher in spindle cell sarcomas but was not significantly different among tumors. In terms of lymph nodes, the Maximum2DDiameterSlice showed a significantly higher value in lymphoma and a significantly lower value in spindle cell sarcoma than in other tumors. The InterauqartileRange exhibited significantly lower values for lymphoma and significantly higher values for spindle cell sarcoma as compared to other tumors.

### 3.9 Comparison among adenocarcinoma, lymphoma, and spindle cell sarcoma in commonly selected CT findings

All commonly selected radiologic findings showed significant differences among tumor types. Lymphomas demonstrated a higher prevalence of concentric growth, whereas spindle cell sarcomas showed a higher prevalence of eccentric growth than other tumors. Adenocarcinomas showed a higher proportion of obstruction and fat stranding than other tumors. Lymphomas exhibited a significantly higher prevalence of homogenous enhancement than other tumors. The results are summarized in Table 3.

**Table 3:** Comparison among commonly selected radiologic findings.

		Adenocarcinoma	Lymphoma	Spindle Cell Sarcoma	*p*-value
Growth pattern	Concentric	2/9 (22.2%)	10/14 (71.4%)	0/19 (0%)	<0.001
	Eccentric	1/9 (11.1%)	4/14 (28.6%)	14/19 (73.7%)	
	Mixed	6/9 (66.7%)	0/14 (0%)	5/19 (26.3%)	
Obstruction	Present	4/9 (44.4%)	1/14 (7.1%)	2/19 (10.5%)	0.048
	Absent	5/9 (55.6%)	13/14 (92.9%)	17/19 (89.5%)	
Fat stranding	Present	6/9 (66.7%)	2/14 (14.3%)	4/19 (21.1%)	0.025
	Absent	3/9 (33.3%)	12/14 (85.7%)	15/19 (78.9%)	

*p < 0.05 is considered statistically significant.*

## 4 Discussion

Precision medicine allows the extraction of various types of clinical information on tumors and has, therefore, emerged as a crucial factor in modern oncology (13, 46). Radiomics is a widely accepted form of precision medicine that extracts quantitative features from clinical images and identifies disease characteristics that can be used as biomarkers (32). Ιt is commonly used in oncology for tumor diagnosis, prognosis, and tumor response (32, 47). For example, it plays an important role in tumor response, prediction, and evaluation of tumor reduction after chemoradiation (27–30). In recent decades, the interest in precision medicine and radiomics in veterinary medicine has markedly increased (48). The radiomics features of various tumors, including those of the lungs, liver, spleen, and adrenal glands, have been investigated (34, 35, 49, 50). In human medicine, there have been many studies on radiomics or gastrointestinal tumor texture analysis. The application of radiomics in colorectal tumors, including the prediction of BRAF mutations, perineural invasion, lung metastasis, and response to chemoradiation, has been extensively studied (26, 51–55). A tumor classification model based on CT tumor texture analysis of gastric tumors showed adequate performance in terms of differentiating adenocarcinoma–lymphoma and lymphoma–gastrointestinal stromal tumors but poor performance in differentiating adenocarcinoma–gastrointestinal stromal tumors (40).

This study tested the performance of multinomial logistic regression, random forest, and SVM models in various environments. Various environments included multiple-bin settings, contrast enhancement, and the inclusion or exclusion of intraluminal gas. We investigated whether the addition of radiologic features interpreted by radiologists affect the performance of radiomics models. Numerous models showed adequate performance; however, the SVM BC16 model using tumor and mesenteric lymph node segmentation in post-contrast images intraluminal gas excluded with radiologic findings showed the best tumor differentiation ability. Thus, this model May be ideal for applications in clinical settings.

In radiomics, four feature-selection methods exist: filter, wrapper, embedded, and dimension reduction (18). Filter methods utilize the most meaningful features by measuring direct associations between features and outcomes (1). Statistical analyses include (but are not limited to) Student’s t-test, correlation coefficient, analysis of variance, and chi-squared tests (56). CT texture analysis of canine splenic tumors uses the Mann–Whitney U test to select meaningful radiomic features (35). However, this method fails to consider collinearity among the selected features, and therefore can be unsuitable (1). The wrapper method utilizes machine-learning models to find optimal features by analyzing possible feature sets; however, it incurs a high computational cost (1). The embedded method selects features during the model-building process, and LASSO and ridge regression are commonly used (1). The dimension reduction method utilizes high-dimensional features in different computational spaces to create a compact representation (1).

Training and test sets were required to validate the created model. The hold-out method uses a training set to create a model and a validation set to test model performance on new data (57). The K-fold cross-validation method divides the data into K-folds and uses K-1 folds to train the model, while the remaining fold is used to validate the model (57). This study used the embedded and hold-out methods, LASSO with 0.3 hold-out ratio with 100 repetitions to select features and divide the training and test sets. LASSO is a commonly used method in human radiomics that theoretically provides maximum orthogonality between features and derives sparse results (1). Therefore, its use for feature selection is highly recommended for veterinary radiomics.

The PT models analyzed only tumor segmentation, whereas the PTLN models incorporated both tumor and mesenteric lymph node segmentations. The PTLN models showed superior performance compared to the PT models in all three types of radiomics models (multinomial logistic regression, random forest, and SVM). This indicated that the incorporation of lymph nodes strongly enhanced the performance of the radiomics models. Thus, for radiomics models differentiating small-intestinal tumors, mesenteric lymph nodes should be included in the dataset. The inclusion of lymph nodes is plausible, because radiologists consider lymph node morphology and size when interpreting small intestinal tumor types (41).

Common models used in radiomics include linear discriminant analysis, multinomial logistic regression, support vector machines, and random forest (21–24). The CT texture analysis of canine hepatic and splenic tumors uses discriminant analysis for model building (34, 35). A study investigating pulmonary parenchymal texture changes in canine pulmonary thromboembolism used an SVM model for classification (58). This study used multinomial logistic regression, random forest, and SVM for model building. In the PTLN models, the performances of multinomial logistic regression, random forest, and SVM models were compared. SVM models utilize a decision boundary to classify the data and predict where the unclassified data fit (21). The multinomial logistic regression model estimates the association between independent and dependent variables using the logarithm of odds of an event as a linear combination (24, 59). The random forest model constructs decision trees using different bootstrap data for classification (22). The SVM models showed superior performance compared to the other two models. Because the SVM model showed the best performance, its application in the classification of canine small intestinal tumors is recommended.

The incorporation of clinical findings into radiomics models has been investigated in human radiomics. Several studies have highlighted the importance of clinically combined radiomics models for different tumors. A radiomics study differentiating pneumonia-like cancer from the pulmonary inflammatory region reported that age, necrosis, and pleural attachment were effective factors for classification (60). In models predicting the response to neoadjuvant chemoradiotherapy followed by total mesorectal excision of rectal tumors, a model with clinical magnetic resonance imaging and radiomics features performed better than a model with clinical features alone (61). Similar results were reported in a gastrointestinal radiomics study. A CT radiomics study of gastrointestinal stromal tumors also showed that a radiomics model with clinical information performed better (38). Similar to human medicine, this study incorporated radiologic findings into radiomics models and compared them to radiomics models without radiologic findings. Both the SVM and random forest models showed better performance when combined with radiologic findings; however, only the SVM model exhibited a significant difference between the clinical and radiomics models. Considering the improvement in model performance, combining radiologic findings with radiomics features when building a model is recommended.

Commonly selected features in the PTLN model with the best performance included Elongation, LeastAxisLength, SurfaceVolumeRatio, LowGrayLevelZoneEmphasis for pre-contrast tumors, Maximum2DDiameterSlice, Kurtosis, and Minimum for pre-contrast lymph nodes. The Elongation indicates the extent to which the length of a volume is greater than its width (62). The LeastAxisLength represents the axis along which the object is least extended, and the SurfaceVolumeRatio indicates how similar the object is to a sphere, with a lower surface volume ratio indicating a spherical shape (62). The LowGrayLevelZoneEmphasis measures the distribution of lower gray-level zones, with higher values indicating a larger proportion of lower gray-level zones (62). The Maximum2DDiameterSlice represents the greatest Euclidean distance between surface mesh vertices in the row-column plane (62). Kurtosis measures the extent to which the intensity distribution peaks, with the minimum representing the lowest intensity present in an object (62). According to these features, spindle cell sarcomas were longer, larger in size, and less sphere-like. By contrast, adenocarcinomas were smaller, and lymphomas resembled spheres to a greater extent than other tumors. Although no significant differences were noted, spindle cell sarcomas had a lower proportion of low-gray-level zones. Spindle cell sarcomas are well known to be larger than other tumors, whereas adenocarcinomas are smaller (41). Although tumor size in conventional CT findings and radiomics features matched well, spindle cell sarcomas showed a lower proportion of low-gray level zones, which was unexpected because spindle cell sarcomas tend to exhibit a large cystic portion (41). Such a disparity May be due to the difference between the low gray level that the radiologist perceives and that distinguished by radiomics. Lymph node size was larger in lymphomas and smaller in spindle cell sarcomas. Lymph nodes in lymphoma displayed a more peaked distribution, whereas in spindle cell sarcoma, they exhibited a flatter peak. The minimum value was higher for adenocarcinomas. Intestinal lymphoma often shows prominent lymph node metastases with an enlarged size, and this characteristic was represented in radiomics. The higher kurtosis of lymph nodes in lymphoma indicated that more pixels were close to the mean, which May reflect the homogenous attenuation of lymph nodes in lymphoma. The radiomics features of lymph nodes in spindle cell sarcomas are cumbersome to interpret because most mesenteric lymph nodes in spindle cell sarcomas did not show lymphadenopathy. These lymph nodes could represent the radiomics features of normal mesenteric lymph nodes rather than the metastatic lymph nodes of spindle cell sarcoma.

Commonly selected features in the PTLN clinical-radiomics model with the best performance included LeastAxisLength, SurfaceVolumeRatio, Kurtosis for post-contrast tumors and Maximum2DDiameterSlice, and InterquartileRange for post-contrast lymph nodes. InterquartileRange represents the spread of the middle half of the data, and the higher value indicates that the central data portion is spread further (62). According to these features, spindle cell sarcomas had a larger size, whereas adenocarcinomas have a smaller size than other tumors. Spindle cell sarcomas were less spherical, whereas lymphomas were more similar to a sphere. Although not statistically significant, spindle cell sarcomas showed higher kurtosis than lymphomas. This contrasts with conventional CT interpretations because lymphomas generally show homogenous enhancement, whereas spindle cell sarcomas exhibit heterogeneous enhancement (41). Lymph nodes in lymphomas were larger and had smaller interquartile ranges than those in other tumors. The smaller interquartile range could be attributed to homogenous lymph nodes, because the central pixel portion was less spread apart. Similar to the PTLN model, radiomics features in the lymph nodes of spindle cell sarcoma should be interpreted with caution because these lymph nodes could represent normal rather than metastatic lymph nodes of spindle cell sarcoma.

Growth pattern, obstruction, and fat stranding were selected as features in the PTLN clinical radiomics model with the best performance. Statistically, the three tumors exhibited different growth patterns. Adenocarcinomas showed a mixed pattern, whereas lymphomas exhibited a concentric pattern. In most cases, spindle cell sarcomas demonstrated an eccentric pattern. Obstruction was less likely to occur in lymphoma and spindle cell sarcomas. Fat stranding was more likely to occur in adenocarcinomas. Tumor growth patterns and the presence or absence of obstruction are key characteristics of differentiating intestinal tumors; therefore, it is not surprising that the radiomics model utilized such findings for model building (41, 42). Fat stranding was an unexpected selection because previous studies reported a low prevalence of adenocarcinoma (41, 42). However, in this study, the prevalence of adenocarcinoma was higher than that reported in previous studies, which May explain why fat stranding was selected for the PTLN clinical-radiomics model.

This study has few limitations. These include the small number of tumors for radiomics model building, absence of histopathologic examination of mesenteric lymph nodes for definitive diagnosis, possibility of mesenteric lymph nodes representing normal lymph nodes instead of metastatic lymph nodes of spindle cell sarcoma, and subjectivity of interpretation of tumor radiologic findings. The amount of data is a critical factor in radiomics model building, and 42 cases May be insufficient for adequate model development. Further inclusion of intestinal tumors would improve the radiomics model reliability. Additionally, although lymph nodes assessed for lymphadenopathy were deemed likely to be metastases of primary intestinal tumors, they were not definitively diagnosed as metastases of intestinal tumors via histopathological examination. Some cases May have involved reactive lymphadenopathy, and the radiomic features of such lymph nodes May have not reflected the intestinal tumor type. In addition, most mesenteric lymph nodes in spindle cell sarcomas did not exhibit lymphadenopathy. Therefore, it was difficult to interpret the lymph node radiomics features in spindle cell sarcoma. Furthermore, due to the retrospective nature and varying patient weights, CT acquisition parameters and time required for contrast administration completion were not harmonized. Contrast enhancement intensity would directly affect the HU, therefore impacting radiomics feature analysis. Lastly, the radiologic assessment incorporated into PTLN clinical radiomics was subjective and could differ depending on the radiologist.

In conclusion, this study demonstrated that the clinical SVM radiomics model of BC16 in post-contrast CT with segmented tumors and mesenteric lymph nodes, but without intraluminal gas, showed the best performance. The inclusion of mesenteric lymph nodes and radiologic findings greatly enhanced the performance of radiomics models. Therefore, the incorporation of mesenteric lymph node segmentation and radiologic findings to build a PTLN clinical-radiomics model is recommended for better model performance.

## Data Availability

The original contributions presented in the study are included in the article/Supplementary material, further inquiries can be directed to the corresponding author/s.

## Footnotes

^1^version 5.2.1; https://www.slicer.org
