# Supplementary material for: Computed tomography radiomics models of tumor differentiation in canine small intestinal tumors
Source: Front Vet Sci. 2024 Sep 23;11:1450304. doi: 10.3389/fvets.2024.1450304 (PMC11457012; doi:10.3389/fvets.2024.1450304)
Supplement: Supplementary file 1 [file Table_1.docx]

Supplementary Table 1. Computed Tomography Acquisition Settings.

|  | CT Scanner | kVp | mAs | Slice thickness (mm) | Helical Pitch  (mm) |
| --- | --- | --- | --- | --- | --- |
| 1 | Lightspeed | 120 | 200 | 2.5 | 1.5 |
| 2 | Revolution ACT | 120-130 | 85-125 | 1.0-2.5 | 1.0-1.3 |
| 3 | Brivo 385 | 120 | 55-100 | 1.25-2.5 | 1.3 |
| 4 | Aquiliion | 120 | 150 | 2.0 | N/A |
| 5 | Aquilion Lightning | 100-12- | 100-180 | 1.0-2.0 | 0.8-0.9 |
| 6 | BrightSpeed | 120 | 71-90 | 1.25 | 1.7 |
| 7 | Somatom Scope | 130 | 120-165 | 0.8-2.0 | 0.8-0.9 |

N/A, Not Available

Supplementary Table 2. Extracted Radiomics Features.

| Category | Features |
| --- | --- |
| Shape | Elongation,  Flatness LeastAxisLength  MajorAxisLength  Maximum2DDiameterColum  Maximum2DDiameterRow Maximum2DDiameterSlice MeshVolume MinorAxisLength  Sphericity  SurfaceArea  SurfaceVolumeRatio  VoxelVolume |
| Firstorder | 10Percentile  90 Percentile  Energy  Entropy  InterquartileRange  Kurtosis  Maximum  MeanAbsoluteDeviation  Mean  Median  Minimum  Range  RobustMeanAbsoluteDeviation  RootMeanSqaured  Skewness  TotalEnergy  Uniformity  Variance |
| GLCM | Autocorrelation  ClusterProimnence  ClusterShade  ClusterTendency  Contrast  Correlation  DifferenceAverage  DifferenceEntropy  DifferenceVariance  Id  Idm  Idmn  Idn  Imc1  Imc2  Inversevariance  JointAverage  JointEntropy  MCC  MaximumProbability  SumAverage  Sumentropy  SumSqaures |
| GLSZM | GrayLevelNonUniformity  GrayLevelNonUniformityNormalized  GrayLevelVariance  HighGrayLevelZoneEmphasis  LargeAreaEmphasis  LargeAreaHighGrayLevelEmphasis  LargeAreaLowGrayLevelEmphasis  LowGrayLevelZoneEmphasis  SizeZoneNonUniformity  SizeZoneNonUniformityNormalized  SmallAreaEmphasis  SmallAreaHighGrayLevelEmphasis  SmallAreaLowGrayLevelEmphasis  ZoneEntropy  ZonePercentage  ZoneVariance |

GLCM, Gray Level Co-occurrence Matrix; GLSZM, Gray Level Size Zone Matrix

Supplementary Table 3. Patient Demographics.

|  | Tumor | Age | Sex | Breed | Body weight |
| --- | --- | --- | --- | --- | --- |
| 1 | Lymphoma | 8 | IF | Shiba Inu | 9.6 |
| 2 | Lymphoma | 9 | SF | Dachshund | 4.6 |
| 3 | Lymphoma | 15 | IF | Pomeranian | 2.6 |
| 4 | Lymphoma | 13 | SF | Poodle | N/A |
| 5 | Lymphoma | 7 | IF | Cane Corso | 37.0 |
| 6 | Lymphoma | 12 | CM | Miniature Pinscher | 4.1 |
| 7 | Lymphoma | 10 | SF | Maltese | N/A |
| 8 | Lymphoma | 6 | SF | Maltese | 3.0 |
| 9 | Lymphoma | 10 | SF | Maltese | 2.8 |
| 10 |  |  |  |  |  |
| 11 | Lymphoma | 15 | SF | Schnauzers | 5.9 |
| 12 | Lymphoma | 14 | SF | Poodle | 3.5 |
| 13 | Lymphoma | 5 | CM | Pug | 11.2 |
| 14 | Lymphoma | 14 | CM | Maltese | 3.4 |
| 15 | Spindle cell sarcoma | 8 | SF | Maltese | 3.2 |
| 16 | Spindle cell sarcoma | 16 | CM | Maltese | N/A |
| 17 | Spindle cell sarcoma | 15 | SF | Yorkshire Terrier | 4.7 |
| 18 | Spindle cell sarcoma | 11 | CM | Pomeranian | N/A |
| 19 | Spindle cell sarcoma | 7 | CM | Maltese | N/A |
| 20 | Spindle cell sarcoma | 7 | CM | Maltese | 6.3 |
| 21 | Spindle cell sarcoma | 8 | CM | Yorkshire Terrier | 2.8 |
| 22 | Spindle cell sarcoma | 10 | CM | Miniature Pinscher | 6.3 |
| 23 | Spindle cell sarcoma | 9 | SF | Beagle | 16.5 |
| 24 | Spindle cell sarcoma | 12 | CM | Poodle | 6.4 |
| 25 | Spindle cell sarcoma | 11 | IF | Rottweiler | 28.7 |
| 26 | Spindle cell sarcoma | 13 | CM | Maltese | 3.0 |
| 27 | Spindle cell sarcoma | 16 | SF | Poodle | 3.3 |
| 28 | Spindle cell sarcoma | 12 | SF | Yorkshire Terrier | 3.2 |
| 29 | Spindle cell sarcoma | 11 | CM | Mixed | 16.3 |
| 30 | Spindle cell sarcoma | 16 | SF | Beagle | N/A |
| 31 | Spindle cell sarcoma | 11 | SF | Spitz | 8.6 |
| 32 | Spindle cell sarcoma | 13 | CM | Maltese | 5.0 |
| 33 | Spindle cell sarcoma | 12 | SF | Pungsan | 20.8 |
| 34 | Adenocarcinoma | 10 | IF | Dachshund | 4.4 |
| 35 | Adenocarcinoma | 15 | SF | Schnauzer | 4.7 |
| 36 | Adenocarcinoma | 12 | SF | Shih Tzu | 6.4 |
| 37 | Adenocarcinoma | 5 | SF | Bichon Frise | 3.2 |
| 38 | Adenocarcinoma | 18 | CM | Maltese | 4.1 |
| 39 | Adenocarcinoma | 12 | IM | Cocker Spaniel | 9.7 |
| 40 | Adenocarcinoma | 8 | SF | Shetland Sheepdog | 8.5 |
| 41 | Adenocarcinoma | 13 | CM | Poodle | 3.0 |
| 42 | Adenocarcinoma | 14 | CM | Maltese | 1.9 |

N/A, Not Available

Supplementary Table 4. Training Accuracy and AUC of Primary Tumor Multinomial Logistic Radiomics Models.

| Bin Settings | Training Accuracy | | | | Training AUC | | | | |
| --- | --- | --- | --- | --- | --- | --- | --- | --- | --- |
|  | Method 1 | Method 2 | Method 3 | Method 4 | | Method 1 | Method 2 | Method 3 | Method 4 |
| BC16 | 0.9229 | 0.9287 | 0.7297 | 0.9642 | | 0.5292 | 0.5775 | 0.5867 | 0.6050 |
| BC32 | 0.9703 | 0.8784 | 0.8755 | 0.9432 | | 0.5775 | 0.5358 | 0.5808 | 0.6583 |
| BC64 | 0.8874 | 0.9694 | 0.8958 | 0.8435 | | 0.5617 | 0.5483 | 0.5642 | 0.5833 |
| BC128 | 0.8987 | 0.8910 | 0.8113 | 0.7884 | | 0.5700 | 0.5092 | 0.5458 | 0.5867 |
| BC256 | 0.8997 | 0.8848 | 0.8061 | 0.7935 | | 0.5758 | 0.5283 | 0.5425 | 0.5700 |
| BW16 | 0.9552 | 1.0000 | 0.8690 | 0.9948 | | 0.5158 | 0.6342 | 0.4992 | 0.6158 |
| BW32 | 0.9181 | 0.8058 | 0.8303 | 0.8384 | | 0.5075 | 0.5417 | 0.4992 | 0.5583 |
| BW64 | 0.9426 | 0.9816 | 0.8303 | 0.7429 | | 0.5342 | 0.6375 | 0.5275 | 0.5300 |
| BW128 | 0.9735 | 0.9303 | 0.9716 | 0.8065 | | 0.6100 | 0.5342 | 0.5658 | 0.5958 |
| BW256 | 0.9723 | 0.8526 | 0.8361 | 0.6884 | | 0.5600 | 0.5725 | 0.5300 | 0.5775 |

BC, Bin Count; BW, Bin Width;

Supplementary Table 5. Test Accuracy and AUC of PT Multinomial Logistic Radiomics Models.

| Bin Settings | Test Accuracy | | | | Test AUC | | | | |
| --- | --- | --- | --- | --- | --- | --- | --- | --- | --- |
|  | Method 1 | Method 2 | Method 3 | Method 4 | | Method 1 | Method 2 | Method 3 | Method 4 |
| BC16 | 0.5292 | 0.5775 | 0.5867 | 0.6050 | | 0.7027 | 0.7741 | 0.7522 | 0.7791 |
| BC32 | 0.5775 | 0.5358 | 0.5808 | 0.6583 | | 0.7451 | 0.7348 | 0.7565 | 0.8224 |
| BC64 | 0.5617 | 0.5483 | 0.5642 | 0.5833 | | 0.7498 | 0.7314 | 0.7344 | 0.7590 |
| BC128 | 0.5700 | 0.5092 | 0.5458 | 0.5867 | | 0.7495 | 0.6973 | 0.7162 | 0.7772 |
| BC256 | 0.5758 | 0.5283 | 0.5425 | 0.5700 | | 0.7487 | 0.7260 | 0.7066 | 0.7659 |
| BW16 | 0.5158 | 0.6342 | 0.4992 | 0.6158 | | 0.6763 | 0.8026 | 0.6801 | 0.8082 |
| BW32 | 0.5075 | 0.5417 | 0.4992 | 0.5583 | | 0.6945 | 0.7278 | 0.6975 | 0.7515 |
| BW64 | 0.5342 | 0.6375 | 0.5275 | 0.5300 | | 0.7181 | 0.8058 | 0.7067 | 0.7416 |
| BW128 | 0.6100 | 0.5342 | 0.5658 | 0.5958 | | 0.7728 | 0.7181 | 0.7535 | 0.8000 |
| BW256 | 0.5600 | 0.5725 | 0.5300 | 0.5775 | | 0.7422 | 0.7249 | 0.7210 | 0.7843 |

BC, Bin Count; BW, Bin Width;

Supplementary Table 6. Training Accuracy and AUC of PTLN Multinomial Logistic Radiomics Models.

| Bin Settings | Training Accuracy | | | | Training AUC | | | | |
| --- | --- | --- | --- | --- | --- | --- | --- | --- | --- |
|  | Method 1 | Method 2 | Method 3 | Method 4 | | Method 1 | Method 2 | Method 3 | Method 4 |
| BC16 | 1.0000 | 1.0000 | 1.0000 | 1.0000 | | 1.0000 | 1.0000 | 1.0000 | 1.0000 |
| BC32 | 1.0000 | 1.0000 | 1.0000 | 1.0000 | | 1.0000 | 1.0000 | 1.0000 | 1.0000 |
| BC64 | 1.0000 | 1.0000 | 1.0000 | 1.0000 | | 1.0000 | 1.0000 | 1.0000 | 1.0000 |
| BC128 | 1.0000 | 1.0000 | 1.0000 | 1.0000 | | 1.0000 | 1.0000 | 1.0000 | 1.0000 |
| BC256 | 1.0000 | 1.0000 | 1.0000 | 1.0000 | | 1.0000 | 1.0000 | 1.0000 | 1.0000 |
| BW16 | 1.0000 | 1.0000 | 1.0000 | 1.0000 | | 1.0000 | 1.0000 | 1.0000 | 1.0000 |
| BW32 | 0.9977 | 1.0000 | 1.0000 | 1.0000 | | 0.9995 | 1.0000 | 1.0000 | 1.0000 |
| BW64 | 0.9974 | 1.0000 | 1.0000 | 1.0000 | | 0.9988 | 1.0000 | 1.0000 | 1.0000 |
| BW128 | 1.0000 | 1.0000 | 1.0000 | 1.0000 | | 1.0000 | 1.0000 | 1.0000 | 1.0000 |
| BW256 | 1.0000 | 1.0000 | 0.9977 | 1.0000 | | 1.0000 | 1.0000 | 0.9994 | 1.0000 |

BC, Bin Count; BW, Bin Width;

Supplementary Table 7. Test Accuracy and AUC of PTLN Multinomial Logistic Radiomics Models.

| Bin Settings | Test Accuracy | | | | Test AUC | | | | |
| --- | --- | --- | --- | --- | --- | --- | --- | --- | --- |
|  | Method 1 | Method 2 | Method 3 | Method 4 | | Method 1 | Method 2 | Method 3 | Method 4 |
| BC16 | 0.6950 | 0.6842 | 0.7192 | 0.7592 | | 0.8518 | 0.8404 | 0.8543 | 0.8838 |
| BC32 | 0.7050 | 0.6650 | 0.7250 | 0.7475 | | 0.8421 | 0.8294 | 0.8665 | 0.8881 |
| BC64 | 0.6925 | 0.6667 | 0.7200 | 0.7150 | | 0.8483 | 0.8506 | 0.8622 | 0.8618 |
| BC128 | 0.6775 | 0.6875 | 0.7275 | 0.7142 | | 0.8347 | 0.8431 | 0.8823 | 0.8546 |
| BC256 | 0.7067 | 0.6975 | 0.7092 | 0.7283 | | 0.8497 | 0.8324 | 0.8584 | 0.8583 |
| BW16 | 0.7017 | 0.7200 | 0.6833 | 0.7758 | | 0.8517 | 0.8668 | 0.8360 | 0.9034 |
| BW32 | 0.7267 | 0.7342 | 0.7525 | 0.7625 | | 0.8587 | 0.8722 | 0.8750 | 0.8933 |
| BW64 | 0.7450 | 0.7450 | 0.7417 | 0.7083 | | 0.8621 | 0.8813 | 0.8704 | 0.8448 |
| BW128 | 0.7150 | 0.6942 | 0.7358 | 0.7067 | | 0.8570 | 0.8544 | 0.8728 | 0.8512 |
| BW256 | 0.7158 | 0.6967 | 0.7058 | 0.6775 | | 0.8685 | 0.8438 | 0.8468 | 0.8368 |

BC, Bin Count; BW, Bin Width;

Supplementary Table 8. Training Accuracy and AUC of PT Random Forest Radiomics Models.

| Bin Settings | Training Accuracy | | | | Training AUC | | | | |
| --- | --- | --- | --- | --- | --- | --- | --- | --- | --- |
|  | Method 1 | Method 2 | Method 3 | Method 4 | | Method 1 | Method 2 | Method 3 | Method 4 |
| BC16 | 1.0000 | 1.0000 | 1.0000 | 1.0000 | | 1.0000 | 1.0000 | 1.0000 | 1.0000 |
| BC32 | 1.0000 | 1.0000 | 0.9997 | 1.0000 | | 1.0000 | 1.0000 | 1.0000 | 1.0000 |
| BC64 | 1.0000 | 1.0000 | 1.0000 | 1.0000 | | 1.0000 | 1.0000 | 1.0000 | 1.0000 |
| BC128 | 1.0000 | 1.0000 | 1.0000 | 1.0000 | | 1.0000 | 1.0000 | 1.0000 | 1.0000 |
| BC256 | 1.0000 | 1.0000 | 1.0000 | 1.0000 | | 1.0000 | 1.0000 | 1.0000 | 1.0000 |
| BW16 | 1.0000 | 1.0000 | 1.0000 | 1.0000 | | 1.0000 | 1.0000 | 1.0000 | 1.0000 |
| BW32 | 1.0000 | 1.0000 | 1.0000 | 1.0000 | | 1.0000 | 1.0000 | 1.0000 | 1.0000 |
| BW64 | 1.0000 | 1.0000 | 1.0000 | 1.0000 | | 1.0000 | 1.0000 | 1.0000 | 1.0000 |
| BW128 | 1.0000 | 1.0000 | 1.0000 | 1.0000 | | 1.0000 | 1.0000 | 1.0000 | 1.0000 |
| BW256 | 1.0000 | 1.0000 | 1.0000 | 1.0000 | | 1.0000 | 1.0000 | 1.0000 | 1.0000 |

BC, Bin Count; BW, Bin Width;

Supplementary Table 9. Test Accuracy and AUC of PT Random Forest Radiomics Models.

| Bin Settings | Test Accuracy | | | | Test AUC | | | | |
| --- | --- | --- | --- | --- | --- | --- | --- | --- | --- |
|  | Method 1 | Method 2 | Method 3 | Method 4 | | Method 1 | Method 2 | Method 3 | Method 4 |
| BC16 | 0.6025 | 0.5600 | 0.4867 | 0.5950 | | 0.7728 | 0.7363 | 0.6899 | 0.7909 |
| BC32 | 0.6017 | 0.5058 | 0.4958 | 0.6633 | | 0.7696 | 0.6885 | 0.7164 | 0.8294 |
| BC64 | 0.5667 | 0.4983 | 0.5417 | 0.5867 | | 0.7398 | 0.6853 | 0.7450 | 0.7729 |
| BC128 | 0.5867 | 0.5025 | 0.5250 | 0.5333 | | 0.7370 | 0.7109 | 0.7117 | 0.7212 |
| BC256 | 0.5483 | 0.5158 | 0.5208 | 0.5342 | | 0.7209 | 0.7264 | 0.7070 | 0.7234 |
| BW16 | 0.5925 | 0.5242 | 0.5692 | 0.5892 | | 0.7404 | 0.7336 | 0.7010 | 0.7797 |
| BW32 | 0.5850 | 0.4892 | 0.5875 | 0.5883 | | 0.7573 | 0.6917 | 0.7214 | 0.7508 |
| BW64 | 0.5850 | 0.5958 | 0.5817 | 0.5433 | | 0.7622 | 0.7689 | 0.7336 | 0.7263 |
| BW128 | 0.5592 | 0.5233 | 0.5783 | 0.5517 | | 0.7128 | 0.7131 | 0.7609 | 0.7742 |
| BW256 | 0.5800 | 0.5075 | 0.5542 | 0.5658 | | 0.7271 | 0.6631 | 0.7171 | 0.7309 |

BC, Bin Count; BW, Bin Width;

Supplementary Table 10. Training Accuracy and AUC of PTLN Random Forest Radiomics Models.

| Bin Settings | Training Accuracy | | | | Training AUC | | | | |
| --- | --- | --- | --- | --- | --- | --- | --- | --- | --- |
|  | Method 1 | Method 2 | Method 3 | Method 4 | | Method 1 | Method 2 | Method 3 | Method 4 |
| BC16 | 1.0000 | 1.0000 | 1.0000 | 1.0000 | | 1.0000 | 1.0000 | 1.0000 | 1.0000 |
| BC32 | 1.0000 | 1.0000 | 1.0000 | 1.0000 | | 1.0000 | 1.0000 | 1.0000 | 1.0000 |
| BC64 | 1.0000 | 1.0000 | 1.0000 | 1.0000 | | 1.0000 | 1.0000 | 1.0000 | 1.0000 |
| BC128 | 1.0000 | 1.0000 | 1.0000 | 1.0000 | | 1.0000 | 1.0000 | 1.0000 | 1.0000 |
| BC256 | 1.0000 | 1.0000 | 1.0000 | 1.0000 | | 1.0000 | 1.0000 | 1.0000 | 1.0000 |
| BW16 | 1.0000 | 1.0000 | 1.0000 | 1.0000 | | 1.0000 | 1.0000 | 1.0000 | 1.0000 |
| BW32 | 1.0000 | 1.0000 | 1.0000 | 1.0000 | | 0.9995 | 1.0000 | 1.0000 | 1.0000 |
| BW64 | 0.9974 | 1.0000 | 1.0000 | 1.0000 | | 0.9988 | 1.0000 | 1.0000 | 1.0000 |
| BW128 | 1.0000 | 1.0000 | 1.0000 | 1.0000 | | 1.0000 | 1.0000 | 1.0000 | 1.0000 |
| BW256 | 1.0000 | 1.0000 | 0.9977 | 1.0000 | | 1.0000 | 1.0000 | 0.9994 | 1.0000 |

BC, Bin Count; BW, Bin Width;

Supplementary Table 11. Test Accuracy and AUC of PTLN Random Forest Models.

| Bin Settings | Test Accuracy | | | | Test AUC | | | | |
| --- | --- | --- | --- | --- | --- | --- | --- | --- | --- |
|  | Method 1 | Method 2 | Method 3 | Method 4 | | Method 1 | Method 2 | Method 3 | Method 4 |
| BC16 | 0.7658 | 0.7525 | 0.7808 | 0.7717 | | 0.9042 | 0.8864 | 0.8767 | 0.9118 |
| BC32 | 0.7725 | 0.7400 | 0.7692 | 0.7750 | | 0.8917 | 0.8849 | 0.8914 | 0.9231 |
| BC64 | 0.7633 | 0.7417 | 0.7608 | 0.7600 | | 0.8819 | 0.8845 | 0.8852 | 0.9113 |
| BC128 | 0.7617 | 0.7508 | 0.7667 | 0.7500 | | 0.8866 | 0.8813 | 0.8802 | 0.9113 |
| BC256 | 0.7858 | 0.7267 | 0.7792 | 0.7333 | | 0.9004 | 0.8834 | 0.8783 | 0.9030 |
| BW16 | 0.7458 | 0.7075 | 0.7575 | 0.7475 | | 0.8683 | 0.8712 | 0.8549 | 0.8925 |
| BW32 | 0.7708 | 0.7558 | 0.7533 | 0.7333 | | 0.8927 | 0.8872 | 0.8771 | 0.8835 |
| BW64 | 0.7833 | 0.7167 | 0.7775 | 0.7175 | | 0.8857 | 0.8841 | 0.8824 | 0.8780 |
| BW128 | 0.7542 | 0.6842 | 0.7425 | 0.6892 | | 0.8785 | 0.8729 | 0.8698 | 0.8782 |
| BW256 | 0.7567 | 0.7342 | 0.7492 | 0.7408 | | 0.8834 | 0.8833 | 0.8495 | 0.8733 |

BC, Bin Count; BW, Bin Width;

Supplementary Table 12. Training Accuracy and AUC of PT SVM Radiomics Models.

| Bin Settings | Training Accuracy | | | | Training AUC | | | | |
| --- | --- | --- | --- | --- | --- | --- | --- | --- | --- |
|  | Method 1 | Method 2 | Method 3 | Method 4 | | Method 1 | Method 2 | Method 3 | Method 4 |
| BC16 | 0.8268 | 0.8371 | 0.7171 | 0.9361 | | 0.9267 | 0.9364 | 0.8540 | 0.9827 |
| BC32 | 0.8768 | 0.8097 | 0.8219 | 0.8671 | | 0.9576 | 0.9156 | 0.9269 | 0.9514 |
| BC64 | 0.8094 | 0.8706 | 0.8158 | 0.7913 | | 0.9225 | 0.9558 | 0.9204 | 0.9128 |
| BC128 | 0.8177 | 0.8271 | 0.7884 | 0.7471 | | 0.9248 | 0.9229 | 0.8965 | 0.8909 |
| BC256 | 0.8200 | 0.8042 | 0.7906 | 0.7545 | | 0.9307 | 0.9169 | 0.8955 | 0.8964 |
| BW16 | 0.8332 | 0.9465 | 0.8077 | 0.8803 | | 0.9349 | 0.9954 | 0.9074 | 0.9705 |
| BW32 | 0.8287 | 0.7871 | 0.7897 | 0.7797 | | 0.9290 | 0.9124 | 0.9022 | 0.9088 |
| BW64 | 0.8681 | 0.9071 | 0.7935 | 0.7219 | | 0.9515 | 0.9752 | 0.9125 | 0.8827 |
| BW128 | 0.9252 | 0.8206 | 0.8303 | 0.7710 | | 0.9850 | 0.9362 | 0.9570 | 0.9154 |
| BW256 | 0.9032 | 0.7719 | 0.7952 | 0.6739 | | 0.9778 | 0.8998 | 0.9131 | 0.8536 |

BC, Bin Count; BW, Bin Width;

Supplementary Table 13. Test Accuracy and AUC of PT SVM Radiomics Models.

| Bin Settings | Test Accuracy | | | | Test AUC | | | | |
| --- | --- | --- | --- | --- | --- | --- | --- | --- | --- |
|  | Method 1 | Method 2 | Method 3 | Method 4 | | Method 1 | Method 2 | Method 3 | Method 4 |
| BC16 | 0.6175 | 0.6342 | 0.6283 | 0.6883 | | 0.7690 | 0.7785 | 0.7856 | 0.8531 |
| BC32 | 0.6775 | 0.6417 | 0.6583 | 0.7600 | | 0.8304 | 0.7708 | 0.8062 | 0.8875 |
| BC64 | 0.6317 | 0.6250 | 0.6275 | 0.6350 | | 0.8081 | 0.7693 | 0.7832 | 0.7980 |
| BC128 | 0.6658 | 0.6067 | 0.6150 | 0.6150 | | 0.8131 | 0.7734 | 0.7685 | 0.7951 |
| BC256 | 0.6675 | 0.6333 | 0.6142 | 0.6133 | | 0.8134 | 0.7822 | 0.7696 | 0.7931 |
| BW16 | 0.6175 | 0.6967 | 0.6100 | 0.6767 | | 0.7596 | 0.8625 | 0.7580 | 0.8583 |
| BW32 | 0.6175 | 0.6025 | 0.6275 | 0.6158 | | 0.7618 | 0.7782 | 0.7706 | 0.7876 |
| BW64 | 0.6550 | 0.6975 | 0.6150 | 0.5792 | | 0.8162 | 0.8481 | 0.7738 | 0.7744 |
| BW128 | 0.6517 | 0.5733 | 0.6150 | 0.6058 | | 0.8197 | 0.7703 | 0.7952 | 0.8028 |
| BW256 | 0.6192 | 0.5917 | 0.5983 | 0.5917 | | 0.7832 | 0.7490 | 0.7613 | 0.8030 |

BC, Bin Count; BW, Bin Width;

Supplementary Table 14. Training Accuracy and AUC of PTLN SVM Radiomics Models.

| Bin Settings | Training Accuracy | | | | Training AUC | | | | |
| --- | --- | --- | --- | --- | --- | --- | --- | --- | --- |
|  | Method 1 | Method 2 | Method 3 | Method 4 | | Method 1 | Method 2 | Method 3 | Method 4 |
| BC16 | 0.9806 | 0.9297 | 0.9784 | 0.9823 | | 0.9991 | 0.9938 | 0.9990 | 0.9978 |
| BC32 | 0.9861 | 0.9542 | 0.9868 | 0.9884 | | 0.9996 | 0.9960 | 0.9995 | 0.9991 |
| BC64 | 0.9690 | 0.9513 | 0.9771 | 0.9494 | | 0.9984 | 0.9968 | 0.9991 | 0.9937 |
| BC128 | 0.9645 | 0.9587 | 0.9726 | 0.9371 | | 0.9978 | 0.9973 | 0.9981 | 0.9901 |
| BC256 | 0.9806 | 0.9558 | 0.9765 | 0.9581 | | 0.9983 | 0.9964 | 0.9990 | 0.9945 |
| BW16 | 0.9745 | 0.9594 | 0.9623 | 0.9794 | | 0.9993 | 0.9969 | 0.9938 | 0.9995 |
| BW32 | 0.9958 | 0.9529 | 0.9965 | 0.9716 | | 0.9997 | 0.9963 | 0.9999 | 0.9974 |
| BW64 | 0.9684 | 0.9729 | 0.9732 | 0.9510 | | 0.9920 | 0.9989 | 0.9983 | 0.9942 |
| BW128 | 0.9977 | 0.9642 | 0.9813 | 0.9394 | | 1.0000 | 0.9963 | 0.9994 | 0.9933 |
| BW256 | 0.9971 | 0.9487 | 0.9894 | 0.9352 | | 1.0000 | 0.9969 | 0.9994 | 0.9876 |

BC, Bin Count; BW, Bin Width;

Supplementary Table 15. Test Accuracy and AUC of Nodal PTLN Radiomics Models.

| Bin Settings | Test Accuracy | | | | Test AUC | | | | |
| --- | --- | --- | --- | --- | --- | --- | --- | --- | --- |
|  | Method 1 | Method 2 | Method 3 | Method 4 | | Method 1 | Method 2 | Method 3 | Method 4 |
| BC16 | 0.7617 | 0.7550 | 0.7908 | 0.7875 | | 0.9118 | 0.9142 | 0.9241 | 0.9209 |
| BC32 | 0.7692 | 0.7483 | 0.8067 | 0.7983 | | 0.9070 | 0.9068 | 0.9309 | 0.9360 |
| BC64 | 0.7267 | 0.7292 | 0.8042 | 0.7750 | | 0.8921 | 0.8977 | 0.9306 | 0.9093 |
| BC128 | 0.7483 | 0.7100 | 0.7792 | 0.7592 | | 0.8932 | 0.8715 | 0.9266 | 0.9057 |
| BC256 | 0.7400 | 0.7308 | 0.7825 | 0.7517 | | 0.8944 | 0.8759 | 0.9234 | 0.8956 |
| BW16 | 0.7458 | 0.7275 | 0.7167 | 0.7708 | | 0.8858 | 0.8820 | 0.8760 | 0.9214 |
| BW32 | 0.7733 | 0.7550 | 0.7642 | 0.7800 | | 0.9124 | 0.9131 | 0.9062 | 0.9222 |
| BW64 | 0.7908 | 0.7458 | 0.7967 | 0.7717 | | 0.9327 | 0.9071 | 0.9312 | 0.8903 |
| BW128 | 0.7708 | 0.7275 | 0.8225 | 0.7433 | | 0.9160 | 0.8918 | 0.9483 | 0.8927 |
| BW256 | 0.8083 | 0.7558 | 0.7892 | 0.7833 | | 0.9395 | 0.9091 | 0.9186 | 0.9110 |

BC, Bin Count; BW, Bin Width;

Supplementary Table 16. Training Accuracy and AUC of PT Random Forest Clinical-radiomics Models.

| Bin Settings | Training Accuracy | | | | Training AUC | | | | |
| --- | --- | --- | --- | --- | --- | --- | --- | --- | --- |
|  | Method 1 | Method 2 | Method 3 | Method 4 | | Method 1 | Method 2 | Method 3 | Method 4 |
| BC16 | 1.0000 | 0.9997 | 0.9981 | 1.0000 | | 1.0000 | 1.0000 | 0.9990 | 1.0000 |
| BC32 | 1.0000 | 0.9997 | 1.0000 | 1.0000 | | 1.0000 | 1.0000 | 1.0000 | 1.0000 |
| BC64 | 0.9997 | 0.9997 | 1.0000 | 1.0000 | | 1.0000 | 1.0000 | 1.0000 | 1.0000 |
| BC128 | 0.9994 | 1.0000 | 1.0000 | 1.0000 | | 1.0000 | 1.0000 | 1.0000 | 1.0000 |
| BC256 | 0.9977 | 1.0000 | 1.0000 | 1.0000 | | 1.0000 | 1.0000 | 1.0000 | 1.0000 |
| BW16 | 1.0000 | 1.0000 | 1.0000 | 1.0000 | | 1.0000 | 1.0000 | 1.0000 | 1.0000 |
| BW32 | 1.0000 | 0.9997 | 1.0000 | 1.0000 | | 1.0000 | 1.0000 | 1.0000 | 1.0000 |
| BW64 | 1.0000 | 1.0000 | 1.0000 | 1.0000 | | 1.0000 | 1.0000 | 1.0000 | 1.0000 |
| BW128 | 0.9977 | 1.0000 | 1.0000 | 1.0000 | | 1.0000 | 1.0000 | 1.0000 | 1.0000 |
| BW256 | 1.0000 | 0.9997 | 1.0000 | 1.0000 | | 1.0000 | 1.0000 | 1.0000 | 1.0000 |

BC, Bin Count; BW, Bin Width;

Supplementary Table 17. Test Accuracy and AUC of PTLN Random Forest Clinical-radiomics Models.

| Bin Settings | Test Accuracy | | | | Test AUC | | | | |
| --- | --- | --- | --- | --- | --- | --- | --- | --- | --- |
|  | Method 1 | Method 2 | Method 3 | Method 4 | | Method 1 | Method 2 | Method 3 | Method 4 |
| BC16 | 0.7583 | 0.6867 | 0.7167 | 0.7300 | | 0.8849 | 0.8524 | 0.8806 | 0.8967 |
| BC32 | 0.7108 | 0.6783 | 0.7367 | 0.7633 | | 0.8577 | 0.8352 | 0.8942 | 0.9200 |
| BC64 | 0.7308 | 0.6967 | 0.7267 | 0.7050 | | 0.8673 | 0.8527 | 0.8970 | 0.8799 |
| BC128 | 0.7383 | 0.7033 | 0.6767 | 0.6975 | | 0.8819 | 0.8559 | 0.8622 | 0.8822 |
| BC256 | 0.7375 | 0.6967 | 0.6675 | 0.7150 | | 0.8853 | 0.8762 | 0.8514 | 0.8816 |
| BW16 | 0.7458 | 0.7067 | 0.6983 | 0.7292 | | 0.8901 | 0.8866 | 0.8538 | 0.9033 |
| BW32 | 0.7483 | 0.6817 | 0.7125 | 0.7067 | | 0.8972 | 0.8523 | 0.8645 | 0.8685 |
| BW64 | 0.7442 | 0.7050 | 0.6992 | 0.7025 | | 0.8931 | 0.8690 | 0.8603 | 0.8671 |
| BW128 | 0.7417 | 0.6875 | 0.7375 | 0.6733 | | 0.8844 | 0.8672 | 0.8847 | 0.8775 |
| BW256 | 0.7592 | 0.6858 | 0.7175 | 0.7075 | | 0.8665 | 0.8544 | 0.8662 | 0.8697 |

BC, Bin Count; BW, Bin Width;

Supplementary Table 18. Training Accuracy and AUC of PTLN Random Forest Clinical-radiomics Models.

| Bin Settings | Training Accuracy | | | | Training AUC | | | | |
| --- | --- | --- | --- | --- | --- | --- | --- | --- | --- |
|  | Method 1 | Method 2 | Method 3 | Method 4 | | Method 1 | Method 2 | Method 3 | Method 4 |
| BC16 | 1.0000 | 1.0000 | 1.0000 | 1.0000 | | 1.0000 | 1.0000 | 1.0000 | 1.0000 |
| BC32 | 1.0000 | 1.0000 | 1.0000 | 1.0000 | | 1.0000 | 1.0000 | 1.0000 | 1.0000 |
| BC64 | 1.0000 | 1.0000 | 1.0000 | 1.0000 | | 1.0000 | 1.0000 | 1.0000 | 1.0000 |
| BC128 | 1.0000 | 1.0000 | 1.0000 | 1.0000 | | 1.0000 | 1.0000 | 1.0000 | 1.0000 |
| BC256 | 1.0000 | 1.0000 | 1.0000 | 1.0000 | | 1.0000 | 1.0000 | 1.0000 | 1.0000 |
| BW16 | 1.0000 | 1.0000 | 1.0000 | 1.0000 | | 1.0000 | 1.0000 | 1.0000 | 1.0000 |
| BW32 | 1.0000 | 1.0000 | 1.0000 | 1.0000 | | 1.0000 | 1.0000 | 1.0000 | 1.0000 |
| BW64 | 1.0000 | 1.0000 | 1.0000 | 1.0000 | | 1.0000 | 1.0000 | 1.0000 | 1.0000 |
| BW128 | 1.0000 | 1.0000 | 1.0000 | 1.0000 | | 1.0000 | 1.0000 | 1.0000 | 1.0000 |
| BW256 | 1.0000 | 1.0000 | 1.0000 | 1.0000 | | 1.0000 | 1.0000 | 1.0000 | 1.0000 |

BC, Bin Count; BW, Bin Width;

Supplementary Table 19. Test Accuracy and AUC of PTLN Random Forest Clinical-radiomics Models.

| Bin Settings | Test Accuracy | | | | Test AUC | | | | |
| --- | --- | --- | --- | --- | --- | --- | --- | --- | --- |
|  | Method 1 | Method 2 | Method 3 | Method 4 | | Method 1 | Method 2 | Method 3 | Method 4 |
| BC16 | 0.7925 | 0.7558 | 0.7817 | 0.7708 | | 0.9290 | 0.9161 | 0.9112 | 0.9286 |
| BC32 | 0.7650 | 0.7333 | 0.7817 | 0.7858 | | 0.9055 | 0.8982 | 0.9206 | 0.9419 |
| BC64 | 0.7883 | 0.7442 | 0.7842 | 0.7633 | | 0.9114 | 0.9050 | 0.9275 | 0.9356 |
| BC128 | 0.7842 | 0.7517 | 0.7725 | 0.7633 | | 0.9136 | 0.9170 | 0.9055 | 0.9270 |
| BC256 | 0.7817 | 0.7442 | 0.7642 | 0.7533 | | 0.9173 | 0.9052 | 0.8965 | 0.9166 |
| BW16 | 0.7650 | 0.7483 | 0.7692 | 0.7883 | | 0.9020 | 0.9028 | 0.8983 | 0.9200 |
| BW32 | 0.7875 | 0.7533 | 0.7675 | 0.7558 | | 0.9206 | 0.9073 | 0.9124 | 0.9148 |
| BW64 | 0.8200 | 0.7483 | 0.8133 | 0.7417 | | 0.9207 | 0.9071 | 0.9078 | 0.9070 |
| BW128 | 0.7850 | 0.7200 | 0.7817 | 0.7150 | | 0.9097 | 0.9084 | 0.9126 | 0.9016 |
| BW256 | 0.7808 | 0.7525 | 0.7658 | 0.7425 | | 0.9139 | 0.9106 | 0.9114 | 0.9100 |

BC, Bin Count; BW, Bin Width;

Supplementary Table 20. Training Accuracy and AUC of PT SVM Clinical-radiomics Models.

| Bin Settings | Training Accuracy | | | | Training AUC | | | | |
| --- | --- | --- | --- | --- | --- | --- | --- | --- | --- |
|  | Method 1 | Method 2 | Method 3 | Method 4 | | Method 1 | Method 2 | Method 3 | Method 4 |
| BC16 | 0.9445 | 0.9074 | 0.9877 | 0.9990 | | 0.9852 | 0.9727 | 0.9996 | 0.9997 |
| BC32 | 0.9390 | 0.9045 | 0.9894 | 0.9984 | | 0.9878 | 0.9740 | 1.0000 | 0.9999 |
| BC64 | 0.9465 | 0.9758 | 0.9865 | 0.9916 | | 0.9864 | 0.9965 | 0.9997 | 0.9990 |
| BC128 | 0.9684 | 0.9426 | 0.9784 | 0.9877 | | 0.9947 | 0.9877 | 0.9987 | 0.9993 |
| BC256 | 0.9526 | 0.9787 | 0.9784 | 0.9871 | | 0.9871 | 0.9981 | 0.9996 | 0.9988 |
| BW16 | 0.9700 | 0.9952 | 0.9652 | 0.9745 | | 0.9969 | 0.9983 | 0.9966 | 0.9963 |
| BW32 | 0.9532 | 0.9210 | 0.9519 | 0.9235 | | 0.9943 | 0.9749 | 0.9949 | 0.9882 |
| BW64 | 0.9494 | 0.9435 | 0.9490 | 0.9252 | | 0.9927 | 0.9891 | 0.9941 | 0.9856 |
| BW128 | 0.9494 | 0.9423 | 0.9761 | 0.9203 | | 0.9880 | 0.9882 | 0.9948 | 0.9834 |
| BW256 | 0.9477 | 0.9361 | 0.9597 | 0.8971 | | 0.9822 | 0.9818 | 0.9891 | 0.9764 |

BC, Bin Count; BW, Bin Width;

Supplementary Table 21. Test Accuracy and AUC of PT SVM Clinical-radiomics Models.

| Bin Settings | Test Accuracy | | | | Test AUC | | | | |
| --- | --- | --- | --- | --- | --- | --- | --- | --- | --- |
|  | Method 1 | Method 2 | Method 3 | Method 4 | | Method 1 | Method 2 | Method 3 | Method 4 |
| BC16 | 0.7358 | 0.6983 | 0.7675 | 0.9058 | | 0.8690 | 0.8574 | 0.8986 | 0.9770 |
| BC32 | 0.7233 | 0.7092 | 0.7708 | 0.8758 | | 0.8677 | 0.8635 | 0.8997 | 0.9519 |
| BC64 | 0.7500 | 0.7533 | 0.7508 | 0.7975 | | 0.8642 | 0.8712 | 0.8892 | 0.9199 |
| BC128 | 0.7600 | 0.7375 | 0.7208 | 0.7842 | | 0.8850 | 0.8844 | 0.8612 | 0.9074 |
| BC256 | 0.7658 | 0.7275 | 0.7092 | 0.7442 | | 0.8933 | 0.8824 | 0.8615 | 0.8908 |
| BW16 | 0.7258 | 0.7767 | 0.7158 | 0.7650 | | 0.8479 | 0.8871 | 0.8498 | 0.8905 |
| BW32 | 0.7258 | 0.6892 | 0.7075 | 0.7292 | | 0.8429 | 0.8533 | 0.8532 | 0.8854 |
| BW64 | 0.7392 | 0.7383 | 0.7292 | 0.7283 | | 0.8637 | 0.8602 | 0.8584 | 0.8857 |
| BW128 | 0.7658 | 0.6975 | 0.7725 | 0.6925 | | 0.8870 | 0.8569 | 0.8792 | 0.8591 |
| BW256 | 0.7750 | 0.6892 | 0.7625 | 0.7092 | | 0.8737 | 0.8507 | 0.8768 | 0.8694 |

BC, Bin Count; BW, Bin Width;

Supplementary Table 22. Training Accuracy and AUC of PTLN SVM Clinical-radiomics Models.

| Bin Settings | Training Accuracy | | | | Training AUC | | | | |
| --- | --- | --- | --- | --- | --- | --- | --- | --- | --- |
|  | Method 1 | Method 2 | Method 3 | Method 4 | | Method 1 | Method 2 | Method 3 | Method 4 |
| BC16 | 0.9974 | 0.9997 | 1.0000 | 1.0000 | | 1.0000 | 1.0000 | 1.0000 | 1.0000 |
| BC32 | 0.9900 | 1.0000 | 1.0000 | 1.0000 | | 1.0000 | 1.0000 | 1.0000 | 1.0000 |
| BC64 | 0.9890 | 0.9994 | 1.0000 | 0.9997 | | 0.9997 | 1.0000 | 1.0000 | 1.0000 |
| BC128 | 0.9987 | 0.9997 | 0.9997 | 1.0000 | | 1.0000 | 1.0000 | 1.0000 | 1.0000 |
| BC256 | 0.9984 | 0.9997 | 1.0000 | 1.0000 | | 1.0000 | 1.0000 | 1.0000 | 1.0000 |
| BW16 | 0.9994 | 1.0000 | 0.9971 | 0.9990 | | 1.0000 | 1.0000 | 1.0000 | 1.0000 |
| BW32 | 0.9974 | 0.9984 | 0.9968 | 0.9990 | | 0.9998 | 1.0000 | 0.9999 | 1.0000 |
| BW64 | 0.9958 | 1.0000 | 0.9984 | 0.9987 | | 0.9996 | 1.0000 | 0.9996 | 1.0000 |
| BW128 | 0.9990 | 0.9984 | 0.9929 | 0.9955 | | 1.0000 | 0.9998 | 1.0000 | 0.9999 |
| BW256 | 0.9971 | 1.0000 | 1.0000 | 0.9987 | | 1.0000 | 1.0000 | 1.0000 | 1.0000 |

BC, Bin Count; BW, Bin Width;

Supplementary Table 23. Test Accuracy and AUC of PTLN SVM Clinical-radiomics Models.

| Bin Settings | Test Accuracy | | | | Test AUC | | | | |
| --- | --- | --- | --- | --- | --- | --- | --- | --- | --- |
|  | Method 1 | Method 2 | Method 3 | Method 4 | | Method 1 | Method 2 | Method 3 | Method 4 |
| BC16 | 0.8233 | 0.8633 | 0.8592 | 0.9008 | | 0.9417 | 0.9536 | 0.9531 | 0.9775 |
| BC32 | 0.8233 | 0.8633 | 0.8733 | 0.9050 | | 0.9401 | 0.9501 | 0.9495 | 0.9741 |
| BC64 | 0.8325 | 0.8533 | 0.8533 | 0.8733 | | 0.9350 | 0.9430 | 0.9461 | 0.9597 |
| BC128 | 0.8375 | 0.8625 | 0.8258 | 0.8425 | | 0.9482 | 0.9581 | 0.9368 | 0.9487 |
| BC256 | 0.8275 | 0.7975 | 0.8283 | 0.8392 | | 0.9454 | 0.9311 | 0.9464 | 0.9516 |
| BW16 | 0.8142 | 0.8483 | 0.7900 | 0.8567 | | 0.9288 | 0.9523 | 0.9262 | 0.9558 |
| BW32 | 0.8242 | 0.8433 | 0.8017 | 0.8550 | | 0.9445 | 0.9498 | 0.9392 | 0.9534 |
| BW64 | 0.8317 | 0.8558 | 0.8200 | 0.8175 | | 0.9565 | 0.9536 | 0.9565 | 0.9444 |
| BW128 | 0.8142 | 0.8200 | 0.8175 | 0.7917 | | 0.9255 | 0.9384 | 0.9396 | 0.9134 |
| BW256 | 0.8275 | 0.8058 | 0.8342 | 0.7792 | | 0.9329 | 0.9374 | 0.9360 | 0.9223 |

BC, Bin Count; BW, Bin Width
